# Supplementary material for: Human autoimmunity at single cell resolution in aplastic anemia before and after effective immunotherapy
Source: Nat Commun. 2025 May 30;16:5048. doi: 10.1038/s41467-025-60213-6 (PMC12125301; doi:10.1038/s41467-025-60213-6)
Supplement: Supplementary file 1 — Supplementary Information [file 41467_2025_60213_MOESM1_ESM.pdf]

Human Autoimmunity at Single Cell Resolution in Aplastic Anemia Before and After Effective Immunotherapy

Zhijie Wu[1,*], Shouguo Gao[1,*], Xingmin Feng[1,*], Haoran Li[1], Nicolas Sompairac[2], Shirin Jamshidi[2], Desmond Choy[2], Rita Antunes Dos Reis[2], Qingyan Gao[1], Sachiko Kajigaya[1], Lemlem Alemu[1], Diego Quinones Raffo[1], Emma M. Groarke[1], Shahram Korasti[2,3,†], Bhavisha Patel[1,†], and Neal S. Young[1,†]

*These authors contributed equally.

†These authors jointly supervised this work.

Supplementary Data

Supplementary Data 1. Patients' characteristics.

Supplementary Data 2. An antibody panel for CyTOF.

Supplementary Data 3. TCR sequences for GLIPH2 analysis.

Supplementary Data 4. Single-cell sequence metrics of single-cell RNA and single-cell TCR/BCR V(D)J Sequencing.

a

EPAG-IST group	
**Patients cohorts, n**	n = 137
Cohort 3	31
Extension	86
Lab explorative	20
**Age (y), mean (range)**	
**Sex (female/male)**	71/66
**Disease severity (SAA/VSAA)**	70/67
**3 month response, n (%)**	
Overall response	99 (72.3)
Complete response	33 (24.1)
Partial response	66 (48.2)
No response	28 (20.4)
N/A	1 (0.7)
Off study	9 (6.6)
**6 month response, n (%)**	
Overall response	109 (80.0)
Complete response	55 (40.0)
Partial response	54 (39.4)
No response	12 (8.8)
Off study	16 (11.7)
**Baseline ANC (K/ul), mean (range)**	0.32473 (0.000-1.930)
**Baseline PLT (K/ul), mean (range)**	8.43 (0-41)
**Baseline ARC (K/ul), mean (range)**	23.71 (2.0-97.1)
**Baseline ALC (K/ul), mean (range)**	1.4022 (0.13-3.98)
**Mean time to follow up, month (range)**	46.9 (4-89.6)

*IST, immunosuppressive therapy; EPAG, eltrombopag; SAA, severe aplastic anemia; VSAA, very severe aplastic anemia; ANC, absolute neutrophil count; PLT, platelet; ARC, absolute reticulocyte count; ALC, absolute lymphocyte count.

b

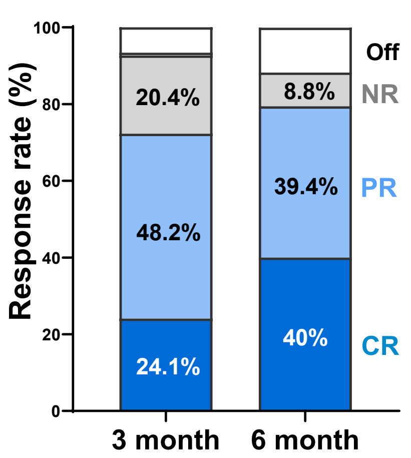

c

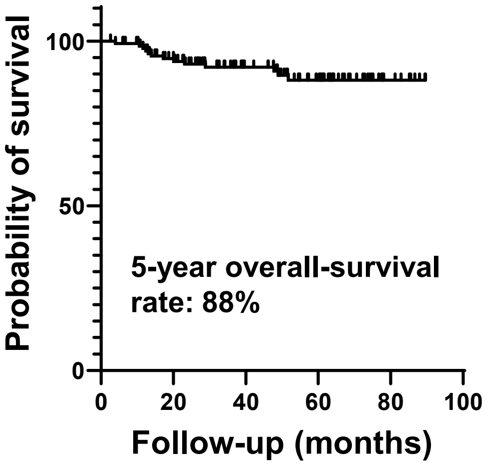

d

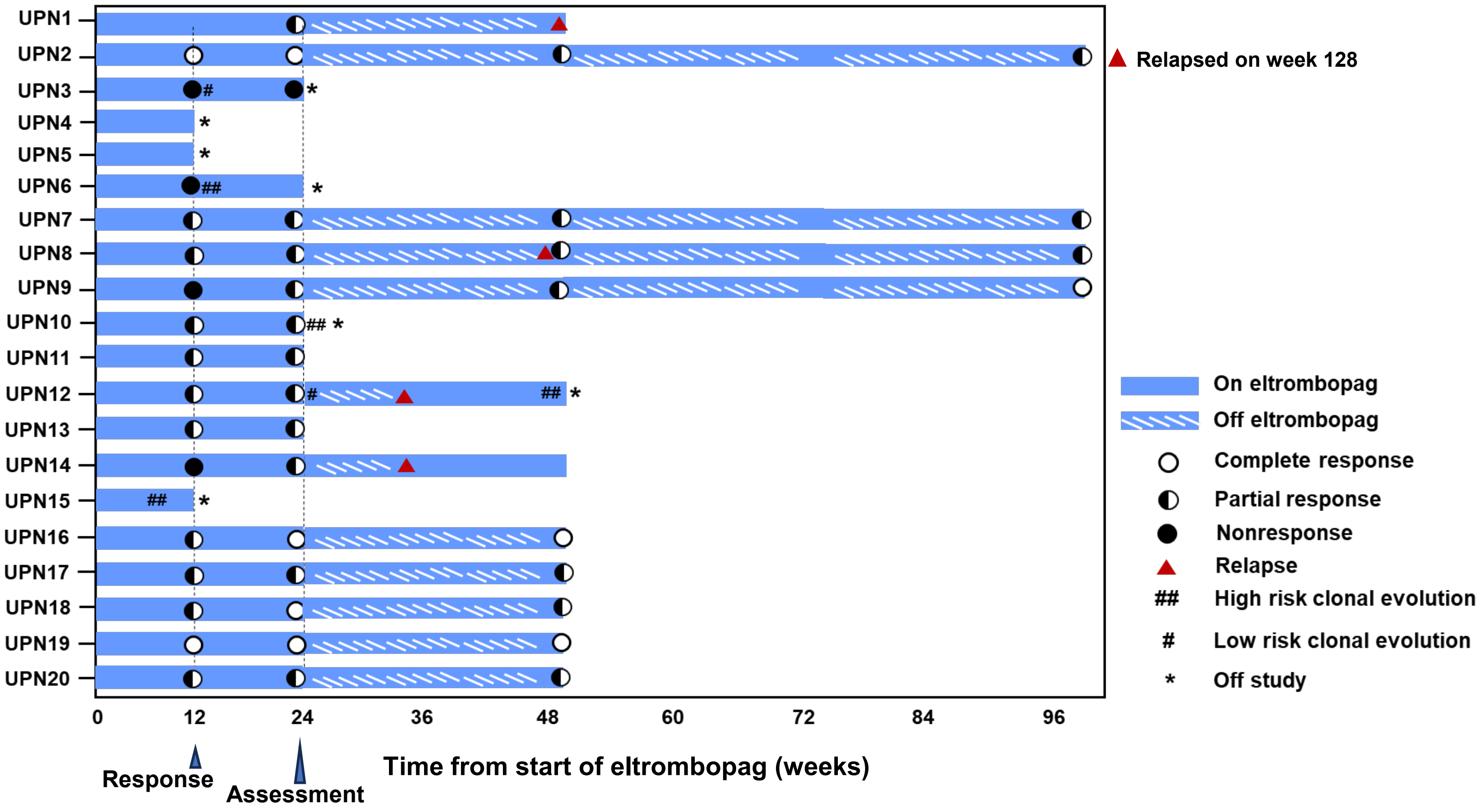

Supplementary Fig. 1 Clinical characteristics of patient cohorts. a, A table showing summary of clinical characteristics of EPAG-IST cohort of patients (*n* = 137), including age, sex, disease severity, responses at 3 months and 6 months after IST, and baseline blood counts. **b**, A bar chart showing response categories (including CR, PR, NR and off study) at 3 months and 6 months after IST. **c**, Kaplan-Meier showing survival of this cohort of patients with medium follow-up time 24.4 months.

d, A Swimmer plot of patient outcomes for a laboratory explorative cohort (*n* = 20). Bars represent treatment and follow-up windows; circles represent responses to treatment and stars designate timepoints at which patients went off-study. EPAG-IST, an eltrombopag-immunosuppressive therapy; CR, complete response; PR, partial response; NR, non-response.

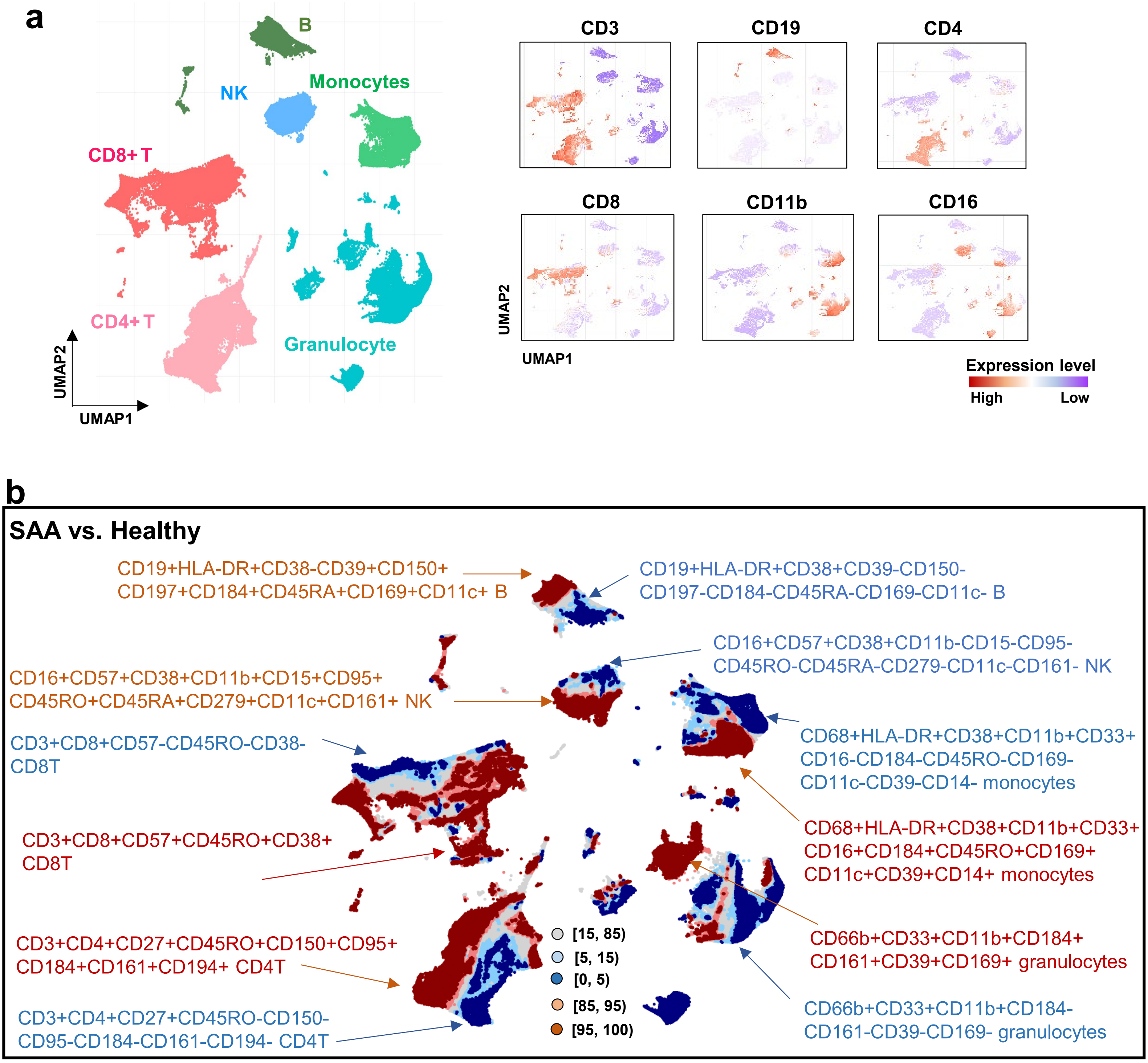

Supplementary Fig. 2 Phenotyping of bone marrow hematopoietic and immune cells using CyTOF in disease. a, Same UMAP of Fig. 1c embedding of CyTOF data based on T-REX analysis, represented cell type specific markers were shown on the right. **b**, Differential abundance and phenotypes of cell populations between SAA patients and healthy donors were compared. A red color indicates clusters with patients' cells (pretreatment samples) constituting >95% of cells and a blue color indicates clusters with healthy donors' cells constituting >95%. For example, CD3^{+}CD8^{+}CD57^{+}CD45RO^{+}CD38^{+}CD8^{+} T cells were increased in patients, while CD3^{+}CD8^{+}CD57^{+}CD45RO^{-}CD38^{-}CD8^{+} T cells were less abundant in patients.

a

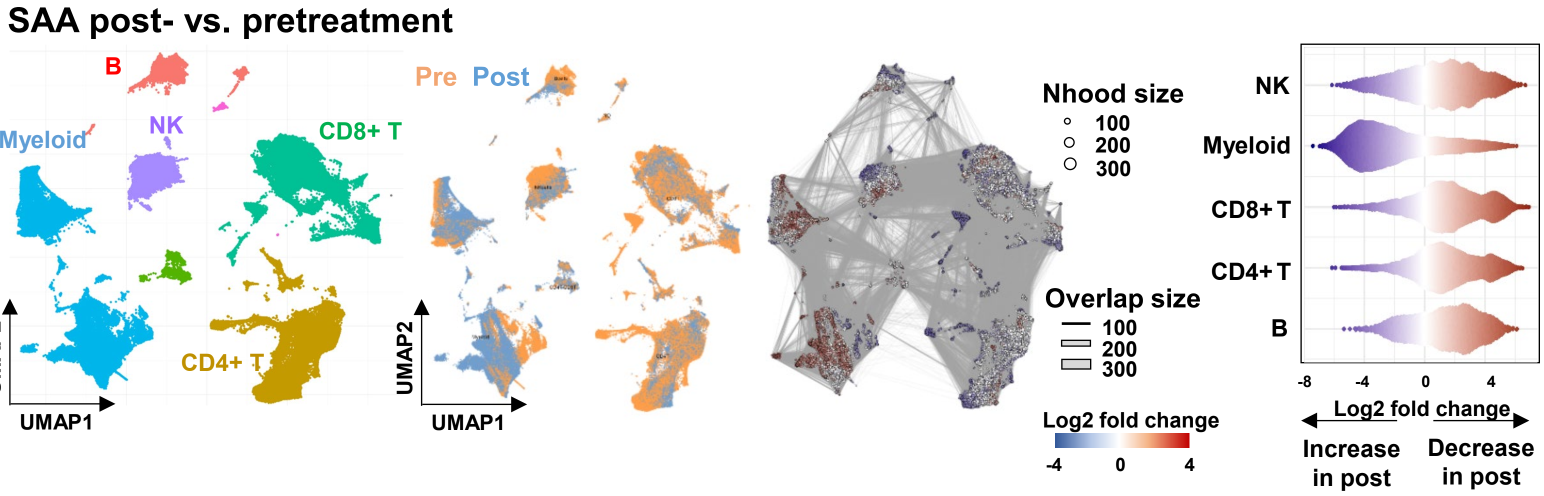

b

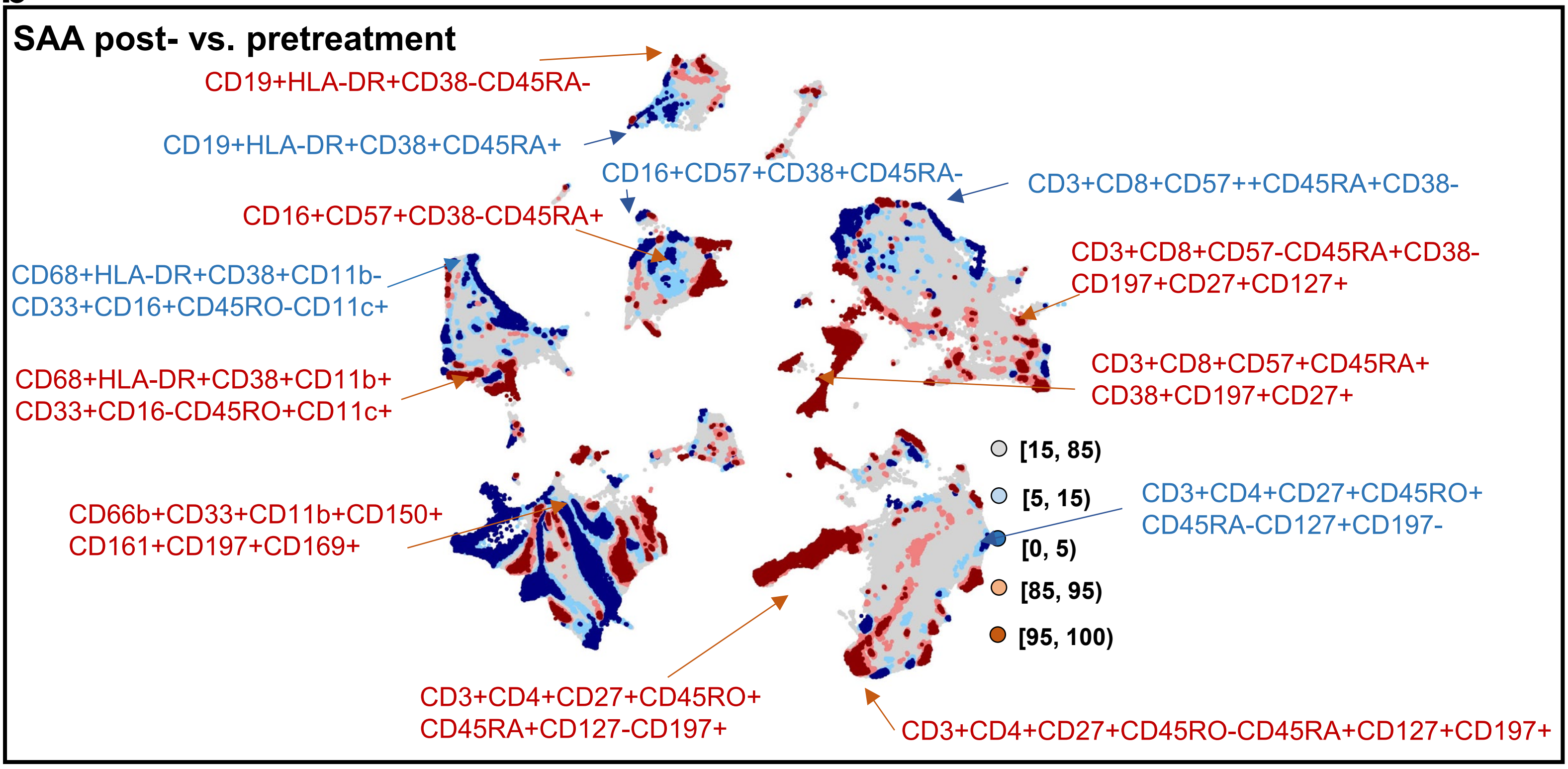

Supplementary Fig. 3 Phenotyping of bone marrow hematopoietic and immune cells using CyTOF before and after treatment. a, Left: UMAP showing differential abundance of cell populations in SAA patients post- versus before-treatment by T-REX. An orange color indicates clusters with patients' cells (posttreatment samples) constituting >95% of cells and a blue color indicates clusters with pretreatment cells constituting >95%. Right: graph representation of Nhoods identified by Milo. Nodes are Nhoods, colored by log2FC between posttreatment samples of SAA patients and pretreatment samples (*n* = 16). Nondifferential abundance Nhoods (FDR ≥ 0.1) are colored white, and sizes correspond to the number of cells in a Nhood. Graph edges depict the number of cells shared between adjacent Nhoods. A Beeswarm plot showing adjusted log2FC of cell population abundance in SAA patients post- versus pretreatment samples in Nhoods. **b,** UMAP showing phenotypes of cell populations in SAA patients post- versus before-treatment by T-REX. For example, $CD68^+HLA\text{-}DR^+CD38^+CD11b^-CD33^+CD16^+CD45RO^-CD11c^+$ myeloid cells decreased while $CD68^+HLA\text{-}DR^+CD38^+CD11b^+CD33^+CD16^-CD45RO^+CD11c^+$ myeloid cells increased after treatment. CyTOF, Cytometry by Time-Of-Flight, UMAP, Uniform Manifold Approximation and Projection; SAA, severe aplastic anemia; log2FC, log2 fold change FDR, false discovery rate.

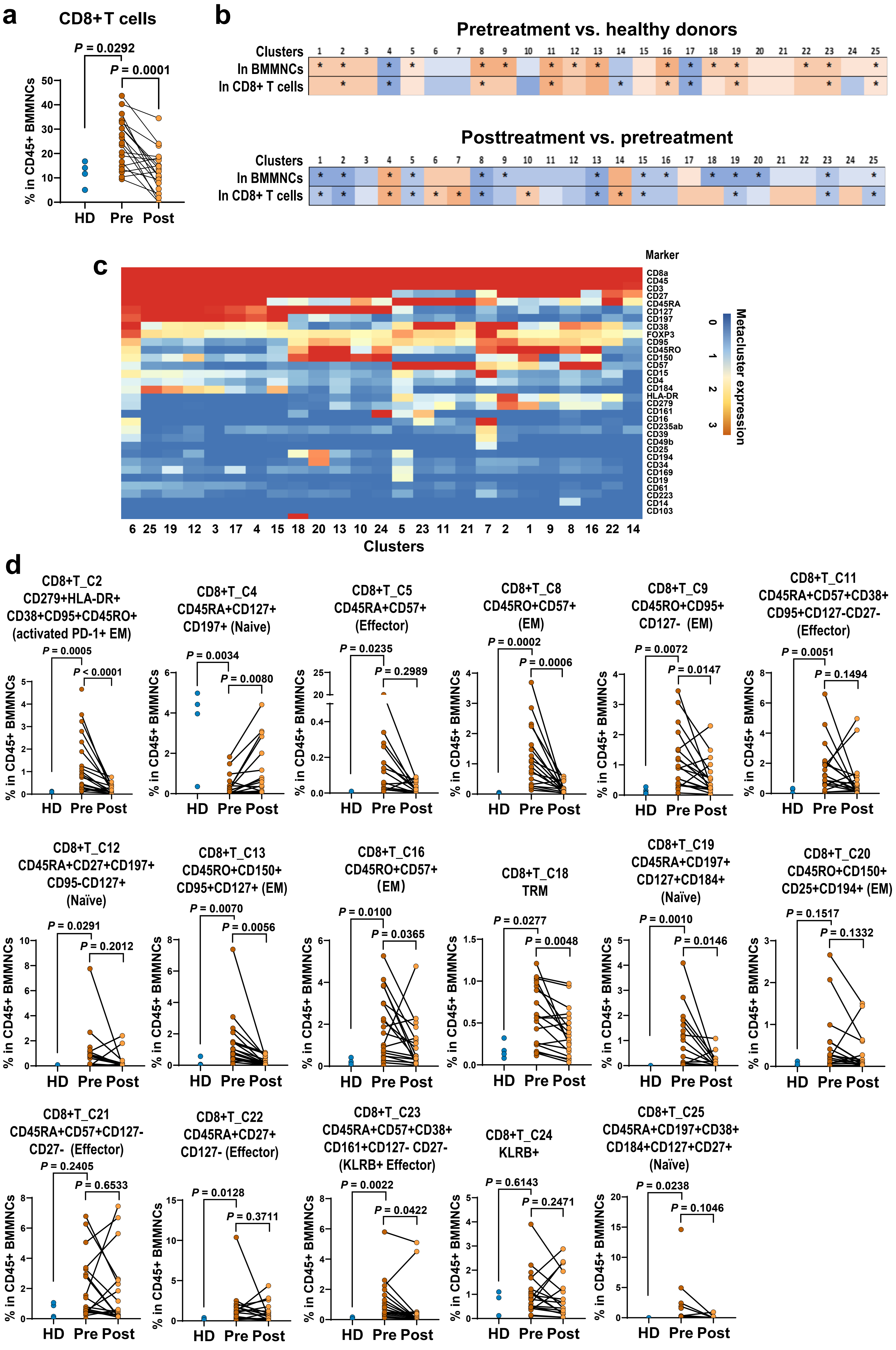
a
CD8+T cells
P = 0.0292
P = 0.0001
% in CD45+ BMMNCs
HD
Pre
Post
b
Pretreatment vs. healthy donors
Clusters
In BMMNCs
In CD8+ T cells
Posttreatment vs. pretreatment
Clusters
In BMMNCs
In CD8+ T cells
c
Marker
CD8a
CD45
CD3
CD27
CD45RA
CD127
CD197
CD38
FOXP3
CD95
CD45RO
CD150
CD57
CD15
CD4
CD184
HLA-DR
CD279
CD161
CD16
CD235ab
CD39
CD49b
CD25
CD194
CD34
CD169
CD19
CD61
CD223
CD14
CD103
Metacluster expression
6 25 19 12 3 17 4 15 18 20 13 10 24 5 23 11 21 7 2 1 9 8 16 22 14
Clusters
d
CD8+T_C2
CD279+HLA-DR+
CD38+CD95+CD45RO+
(activated PD-1+ EM)
P = 0.0005
P < 0.0001
CD8+T_C4
CD45RA+CD127+
CD197+ (Naive)
P = 0.0034
P = 0.0080
CD8+T_C5
CD45RA+CD57+
(Effector)
P = 0.0235
P = 0.2989
CD8+T_C8
CD45RO+CD57+
(EM)
P = 0.0002
P = 0.0006
CD8+T_C9
CD45RO+CD95+
CD127- (EM)
P = 0.0072
P = 0.0147
CD8+T_C11
CD45RA+CD57+CD38+
CD95+CD127-CD27-
(Effector)
P = 0.0051
P = 0.1494
CD8+T_C12
CD45RA+CD27+CD197+
CD95-CD127+
(Naïve)
P = 0.0291
P = 0.2012
CD8+T_C13
CD45RO+CD150+
CD95+CD127+ (EM)
P = 0.0070
P = 0.0056
CD8+T_C16
CD45RO+CD57+
(EM)
P = 0.0100
P = 0.0365
CD8+T_C18
TRM
P = 0.0277
P = 0.0048
CD8+T_C19
CD45RA+CD197+
CD127+CD184+
(Naïve)
P = 0.0010
P = 0.0146
CD8+T_C20
CD45RO+CD150+
CD25+CD194+ (EM)
P = 0.1517
P = 0.1332
CD8+T_C21
CD45RA+CD57+CD127-
CD27- (Effector)
P = 0.2405
P = 0.6533
CD8+T_C22
CD45RA+CD27+
CD127- (Effector)
P = 0.0128
P = 0.3711
CD8+T_C23
CD45RA+CD57+CD38+
CD161+CD127- CD27-
(KLRB+ Effector)
P = 0.0022
P = 0.0422
CD8+T_C24
KLRB+
P = 0.6143
P = 0.2471
CD8+T_C25
CD45RA+CD197+CD38+
CD184+CD127+CD27+
(Naïve)
P = 0.0238
P = 0.1046
% in CD45+ BMMNCs
HD Pre Post

Supplementary Fig. 4 Phenotyping of $CD8^+$ T cell subclusters using CyTOF. a, A scatter plot showing frequency (% in $CD45^+$ BMMNCs) of $CD8^+$ T cells of healthy controls (n = 4), pretreatment samples of SAA patients (n = 20) and posttreatment samples (n = 16). P values with the two-sided unpaired and paired Mann-Whitney test are shown. **b**, Heatmaps showing difference of frequency of 25 clusters of $CD8^+$ T cells in pretreatment samples versus healthy donors (top) and in posttreatment samples versus pretreatment samples (bottom). A red color indicates fold changes to be higher than 1, and a blue color indicates fold changes to be lower than 1. Stars present statistical significance ($P < 0.05$; P values were calculated with the two-sided unpaired Mann-Whitney test). **c**, Binary clustering of 25 clusters of $CD8^+$ T cells based on surface marker expression. Relative expression bars are shown on the right. **d**, Scatter plots showing frequency (% in $CD45^+$ BMMNCs) of representative $CD8^+$ T clusters of healthy controls (n = 4), pretreatment samples of SAA patients (n = 20) and posttreatment samples (n = 16). P values with the two-sided unpaired and paired Mann-Whitney test are shown. Many clusters had differential abundance, for example, $CD8^+$T_C8 and $CD8^+$T_C16 [$CD45RO^+CD57^+$, an effector memory (EM) phenotype] was significantly higher in pretreatment samples as compared to healthy donors, and dramatically decreased after treatment, and $CD8^+$T_C2 ($CD279^+$HLA-$DR^+CD38^+CD95^+CD45RO^+$ activated PD-1^+ EM phenotype) was higher in pretreatment samples and decreased after treatment. BMMNCs, bone marrow mononuclear cells.

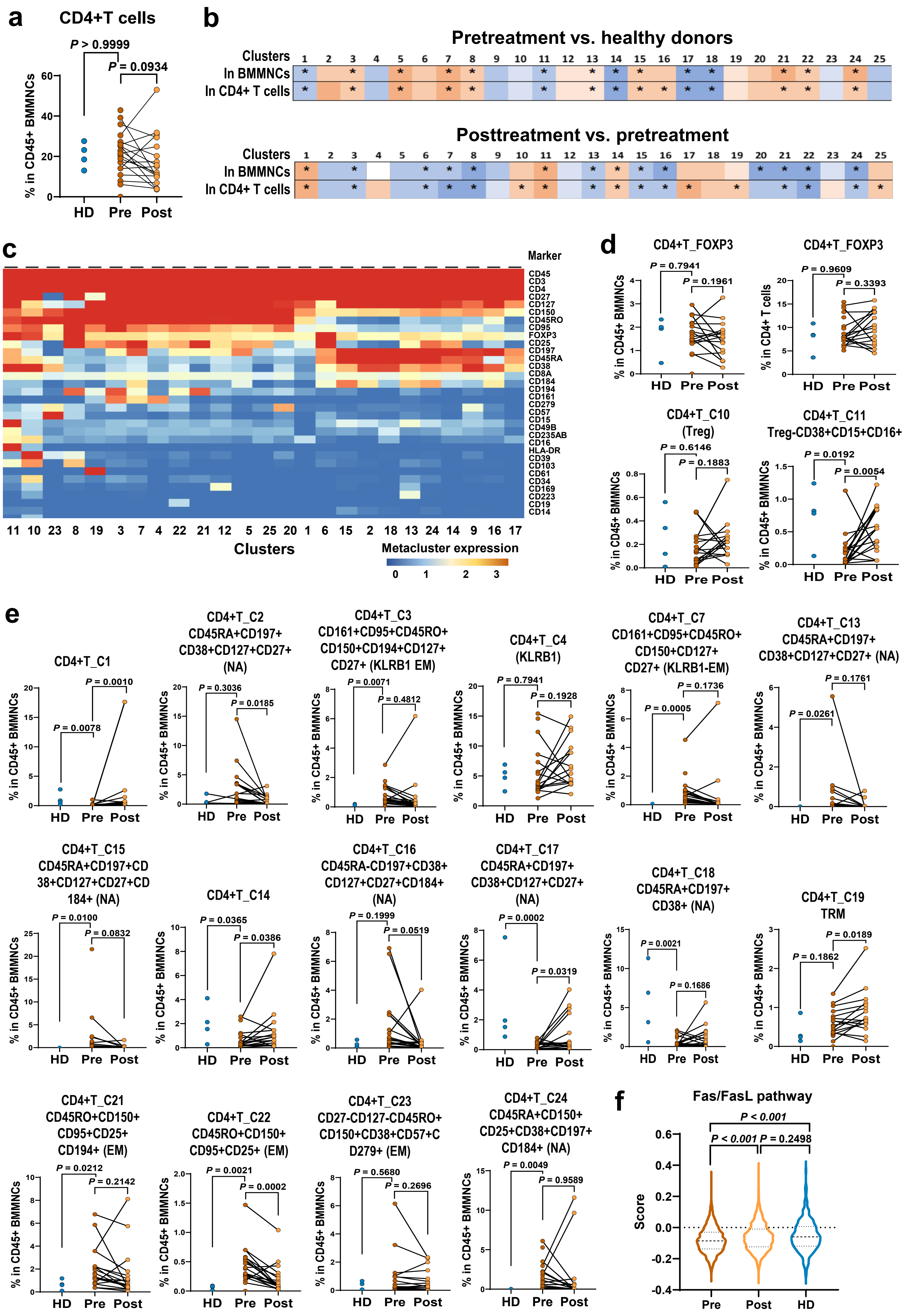

a
CD4+T cells
P > 0.9999
P = 0.0934
% in CD45+ BMMNCs
HD Pre Post
b
Pretreatment vs. healthy donors
Clusters
In BMMNCs
In CD4+ T cells
Posttreatment vs. pretreatment
Clusters
In BMMNCs
In CD4+ T cells
c
Marker
CD45
CD3
CD4
CD27
CD127
CD150
CD45RO
CD95
FOXP3
CD25
CD197
CD45RA
CD38
CD8A
CD184
CD194
CD161
CD279
CD57
CD15
CD49B
CD235AB
CD16
HLA-DR
CD39
CD103
CD61
CD34
CD169
CD223
CD19
CD14
11 10 23 8 19 3 7 4 22 21 12 5 25 20 1 6 15 2 18 13 24 14 9 16 17
Clusters
Metacluster expression
0 1 2 3
d
CD4+T_FOXP3
P = 0.7941
P = 0.1961
% in CD45+ BMMNCs
CD4+T_FOXP3
P = 0.9609
P = 0.3393
% in CD4+ T cells
CD4+T_C10 (Treg)
P = 0.6146
P = 0.1883
CD4+T_C11 Treg-CD38+CD15+CD16+
P = 0.0192
P = 0.0054
e
CD4+T_C1
P = 0.0010
P = 0.0078
CD4+T_C2 CD45RA+CD197+ CD38+CD127+CD27+ (NA)
P = 0.3036
P = 0.0185
CD4+T_C3 CD161+CD95+CD45RO+ CD150+CD194+CD127+ CD27+ (KLRB1 EM)
P = 0.0071
P = 0.4812
CD4+T_C4 (KLRB1)
P = 0.7941
P = 0.1928
CD4+T_C7 CD161+CD95+CD45RO+ CD150+CD127+ CD27+ (KLRB1-EM)
P = 0.1736
P = 0.0005
CD4+T_C13 CD45RA+CD197+ CD38+CD127+CD27+ (NA)
P = 0.1761
P = 0.0261
CD4+T_C15 CD45RA+CD197+CD 38+CD127+CD27+CD 184+ (NA)
P = 0.0100
P = 0.0832
CD4+T_C14
P = 0.0365
P = 0.0386
CD4+T_C16 CD45RA-CD197+CD38+ CD127+CD27+CD184+ (NA)
P = 0.1999
P = 0.0519
CD4+T_C17 CD45RA+CD197+ CD38+CD127+CD27+ (NA)
P = 0.0002
P = 0.0319
CD4+T_C18 CD45RA+CD197+ CD38+ (NA)
P = 0.0021
P = 0.1686
CD4+T_C19 TRM
P = 0.0189
P = 0.1862
CD4+T_C21 CD45RO+CD150+ CD95+CD25+ CD194+ (EM)
P = 0.0212
P = 0.2142
CD4+T_C22 CD45RO+CD150+ CD95+CD25+ (EM)
P = 0.0021
P = 0.0002
CD4+T_C23 CD27-CD127-CD45RO+ CD150+CD38+CD57+C D279+ (EM)
P = 0.5680
P = 0.2696
CD4+T_C24 CD45RA+CD150+ CD25+CD38+CD197+ CD184+ (NA)
P = 0.0049
P = 0.9589
% in CD45+ BMMNCs
HD Pre Post
f
Fas/FasL pathway
P < 0.001
P < 0.001
P = 0.2498
Score
Pre Post HD

Supplementary Fig. 5 Phenotyping of CD4⁺ T cell subclusters using CyTOF. a, A scatter plot showing frequency (% in $CD45^+$ BMMNCs) of $CD4^+$ T cells of healthy controls (*n* = 4), pretreatment samples of SAA patients (*n* = 20) and posttreatment samples (*n* = 16). *P* values with the two-sided unpaired and paired Mann-Whitney test are shown. **b**, Heatmaps showing difference of frequency of 25 $CD4^+$ T cell clusters in pretreatment samples versus healthy donors (top), and in posttreatment samples versus pretreatment samples (bottom). A red color indicates fold change to be higher than 1, and a blue color indicates fold change to be lower than 1. Stars present statistical significance ($P < 0.05$; *P* values were calculated with the two-sided unpaired Mann-Whitney test). **c**, Binary clustering of 25 clusters of $CD4^+$ T cells based on surface marker expression. A relative expression bar is shown on the bottom. **d**, Scatter plots showing frequency (% in $CD45^+$ BMMNCs) of $CD4^+$ Treg clusters of healthy controls (*n* = 4), pretreatment samples of SAA patients (*n* = 20) and posttreatment samples (*n* = 16). *P* values with the two-sided unpaired and paired Mann-Whitney test are shown. **e**, Scatter plots showing frequency (% in $CD45^+$ BMMNCs) of representative $CD4^+$ T clusters of healthy controls (*n* = 4), pretreatment samples of SAA patients (*n* = 20) and posttreatment samples (*n* = 16). *P* values with the two-sided unpaired and paired Mann-Whitney test are shown. **f**, A dot plot showing Fas/FasL pathway scores of CD4+ Tregs in pre- (n = 20), post-treatment (n = 16) samples of SAA patients and healthy controls (n = 4). *P* values with the two-sided unpaired Mann-Whitney test are shown.

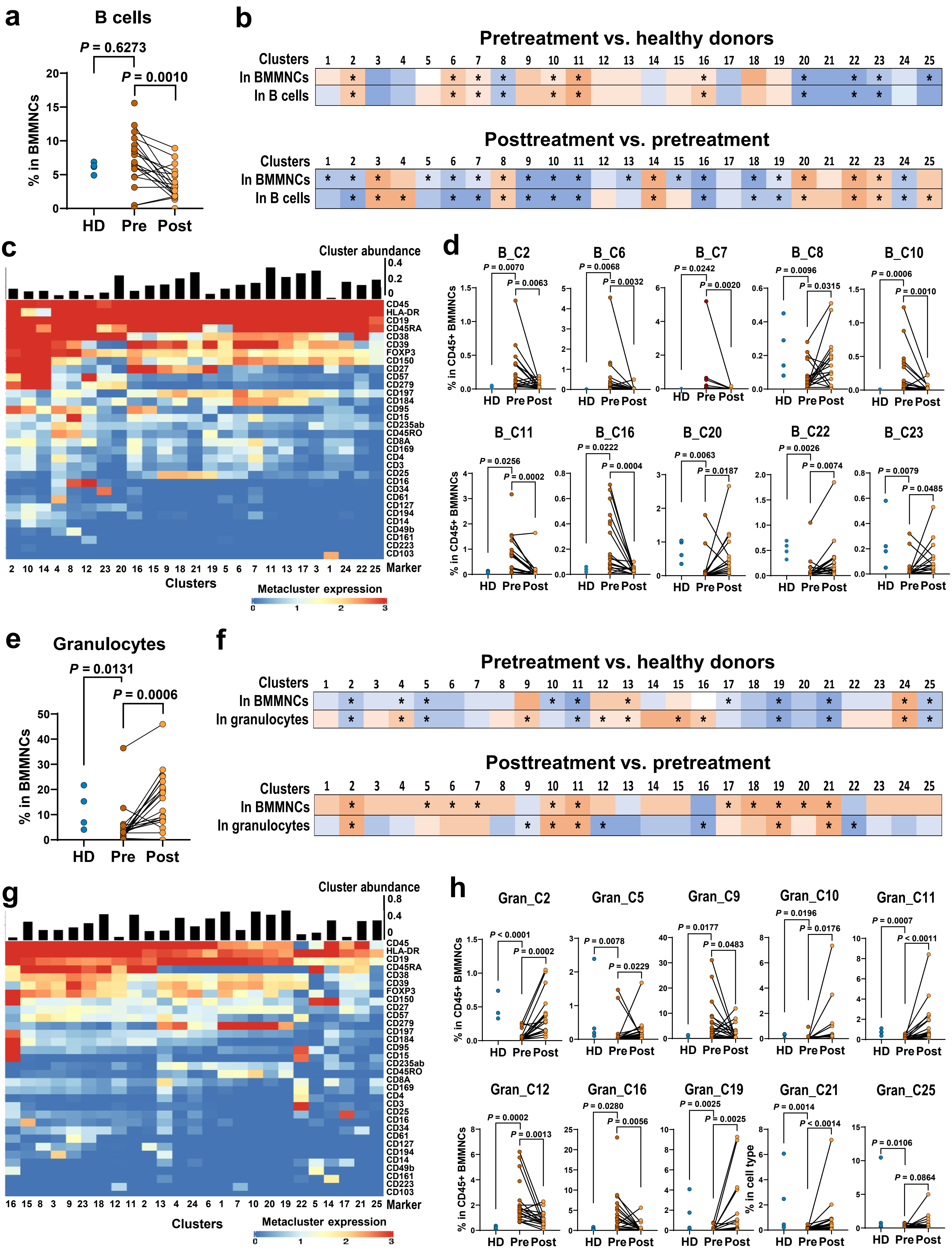

Supplementary Fig. 6 Phenotyping of B cell and granulocyte subclusters using CyTOF. a, A scatter plot showing frequency (% in CD45⁺ BMMNCs) of B cells of HD (*n* = 4), pretreatment (*n* = 20) and posttreatment samples (*n* = 16). *P* values with the two-sided unpaired and paired Mann-Whitney test are shown. **b**, Heatmaps showing difference of frequency of 25 B cell clusters in pretreatment samples versus HD (top) and in posttreatment versus pretreatment samples (bottom). Red color indicates fold change to

be higher than 1, and blue color indicates fold change to be lower than 1. Stars present statistical significance ($P < 0.05$; P values were calculated with the two-sided unpaired Mann-Whitney test). **c**, Binary clustering of 25 clusters of B cells based on surface marker expression. A relative expression bar is shown on the bottom. **d**, Scatter plots showing frequency (% in $CD45^+$ BMMNCs) of representative B clusters of HD (n = 4), pretreatment (n = 20) and posttreatment samples (n = 16). P values with the two-sided unpaired and paired Mann-Whitney test are shown. **e,** A scatter plot showing frequency (% in $CD45^+$ BMMNCs) of granulocytes of healthy controls (n = 4), pretreatment (n = 20) and posttreatment samples (n = 16). P values with the two-sided unpaired and paired Mann-Whitney test are shown. **f**, Heatmaps showing difference of frequency of 25 granulocyte clusters in pretreatment samples versus HD (top) and in posttreatment samples versus pretreatment samples (bottom). A red color indicates fold change to be higher than 1, and a blue color indicates fold change to be lower than 1. Stars present statistical significance ($P < 0.05$; P values were calculated with the two-sided unpaired Mann-Whitney test). **g**, Binary clustering of 25 clusters of granulocytes based on surface marker expression. A relative expression bar is shown on the bottom. **h**, Scatter plots showing frequency (% in $CD45^+$ BMMNCs) of representative granulocyte clusters of HD (n = 4), pretreatment (n = 20) and posttreatment samples (n = 16). P values with the two-sided unpaired and paired Mann-Whitney test are shown.

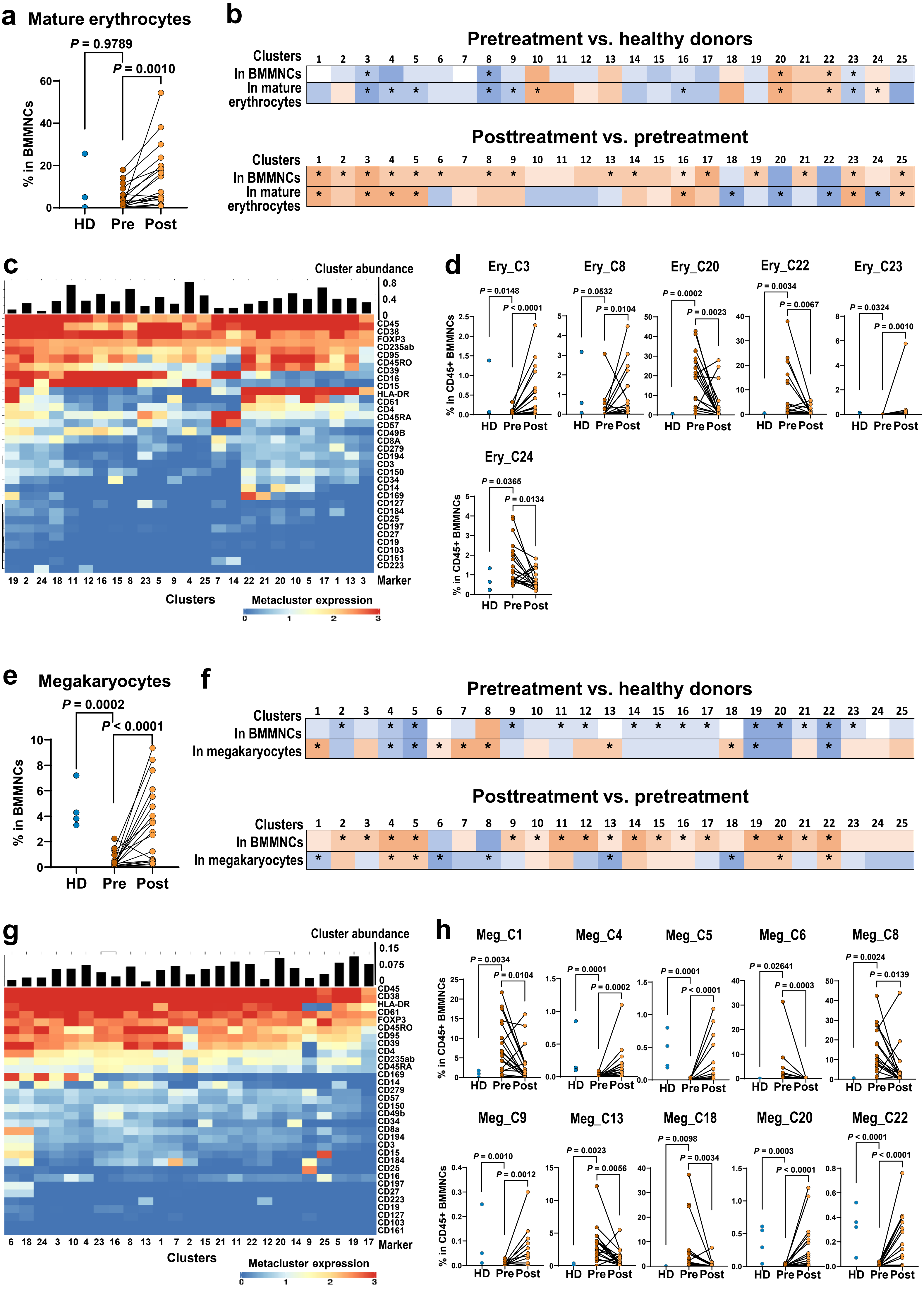
a
Mature erythrocytes
P = 0.9789
P = 0.0010
% in BMMNCs
HD Pre Post
b
Pretreatment vs. healthy donors
Clusters
In BMMNCs
In mature erythrocytes
Posttreatment vs. pretreatment
Clusters
In BMMNCs
In mature erythrocytes
c
Cluster abundance
CD45 CD38 FOXP3 CD235ab CD95 CD45RO CD39 CD16 CD15 HLA-DR CD61 CD4 CD45RA CD57 CD49B CD8A CD279 CD194 CD3 CD150 CD34 CD14 CD169 CD127 CD184 CD25 CD197 CD27 CD19 CD103 CD161 CD223
19 2 24 18 11 12 16 15 8 23 5 9 4 25 7 14 22 21 20 10 5 17 1 13 3
Marker
Clusters
Metacluster expression
d
Ery_C3
P = 0.0148
P < 0.0001
Ery_C8
P = 0.0532
P = 0.0104
Ery_C20
P = 0.0002
P = 0.0023
Ery_C22
P = 0.0034
P = 0.0067
Ery_C23
P = 0.0324
P = 0.0010
Ery_C24
P = 0.0365
P = 0.0134
% in CD45+ BMMNCs
HD Pre Post
e
Megakaryocytes
P = 0.0002
P < 0.0001
% in BMMNCs
HD Pre Post
f
Pretreatment vs. healthy donors
Clusters
In BMMNCs
In megakaryocytes
Posttreatment vs. pretreatment
Clusters
In BMMNCs
In megakaryocytes
g
Cluster abundance
CD45 CD38 HLA-DR CD61 FOXP3 CD45RO CD95 CD39 CD4 CD235ab CD45RA CD169 CD14 CD279 CD57 CD150 CD49b CD34 CD8a CD194 CD3 CD15 CD184 CD25 CD16 CD197 CD27 CD223 CD19 CD127 CD103 CD161
6 18 24 3 10 4 23 16 8 13 1 7 2 15 21 11 22 12 20 14 9 25 5 19 17
Marker
Clusters
Metacluster expression
h
Meg_C1
P = 0.0034
P = 0.0104
Meg_C4
P = 0.0001
P = 0.0002
Meg_C5
P = 0.0001
P < 0.0001
Meg_C6
P = 0.02641
P = 0.0003
Meg_C8
P = 0.0024
P = 0.0139
Meg_C9
P = 0.0010
P = 0.0012
Meg_C13
P = 0.0023
P = 0.0056
Meg_C18
P = 0.0098
P = 0.0034
Meg_C20
P = 0.0003
P < 0.0001
Meg_C22
P < 0.0001
P < 0.0001
% in CD45+ BMMNCs
HD Pre Post

Supplementary Fig. 7 Phenotyping of mature erythrocyte and megakaryocyte subclusters using CyTOF. a, A scatter plot showing frequency (% in $CD45^+$ BMMNCs) of mature erythrocytes of HD (n = 4), pretreatment (n = 20) and posttreatment samples (n = 16). P values with the two-sided unpaired and paired Mann-Whitney test are shown. **b**, Heatmaps showing difference of frequency of 25 mature erythrocyte clusters in pretreatment samples versus healthy donors (top) and in posttreatment versus pretreatment samples (bottom). Red color indicates fold change to be higher than 1, and blue color indicates fold change to be lower than 1. Stars present statistical significance ($P < 0.05$; P values were calculated with the two-sided unpaired Mann-Whitney test). **c**, Binary clustering of 25 clusters of mature erythrocytes based on surface marker expression. A relative expression bar is shown on the bottom. **d**, Scatter plots showing frequency (% in $CD45^+$ BMMNCs) of representative mature erythrocyte clusters of HD (n = 4), pretreatment (n = 20) and posttreatment samples (n = 16). P values with the two-sided unpaired and paired Mann-Whitney test are shown. **e,** A scatter plot showing frequency (% in $CD45^+$ BMMNCs) of megakaryocytes of HD (n = 4), pretreatment (n = 20) and posttreatment samples (n = 16). P values with the two-sided unpaired and paired Mann-Whitney test are shown. **f**, Heatmaps showing difference of frequency of 25 megakaryocyte clusters in pretreatment samples versus HD (top) and in posttreatment versus pretreatment samples (bottom). A red color indicates fold change to be higher than 1, and a blue color indicates fold change to be lower than 1. Stars present statistical significance ($P < 0.05$; P values were calculated with the two-sided unpaired Mann-Whitney test). **g**, Binary clustering of 25 clusters of megakaryocytes based on surface marker expression. A relative expression bar is shown on the right. **h**, Scatter plots showing frequency (% in $CD45^+$ BMMNCs) of representative megakaryocyte clusters of HD (n = 4), pretreatment (n = 20) and posttreatment samples (n = 16). P values with the two-sided unpaired and paired Mann-Whitney test are shown.

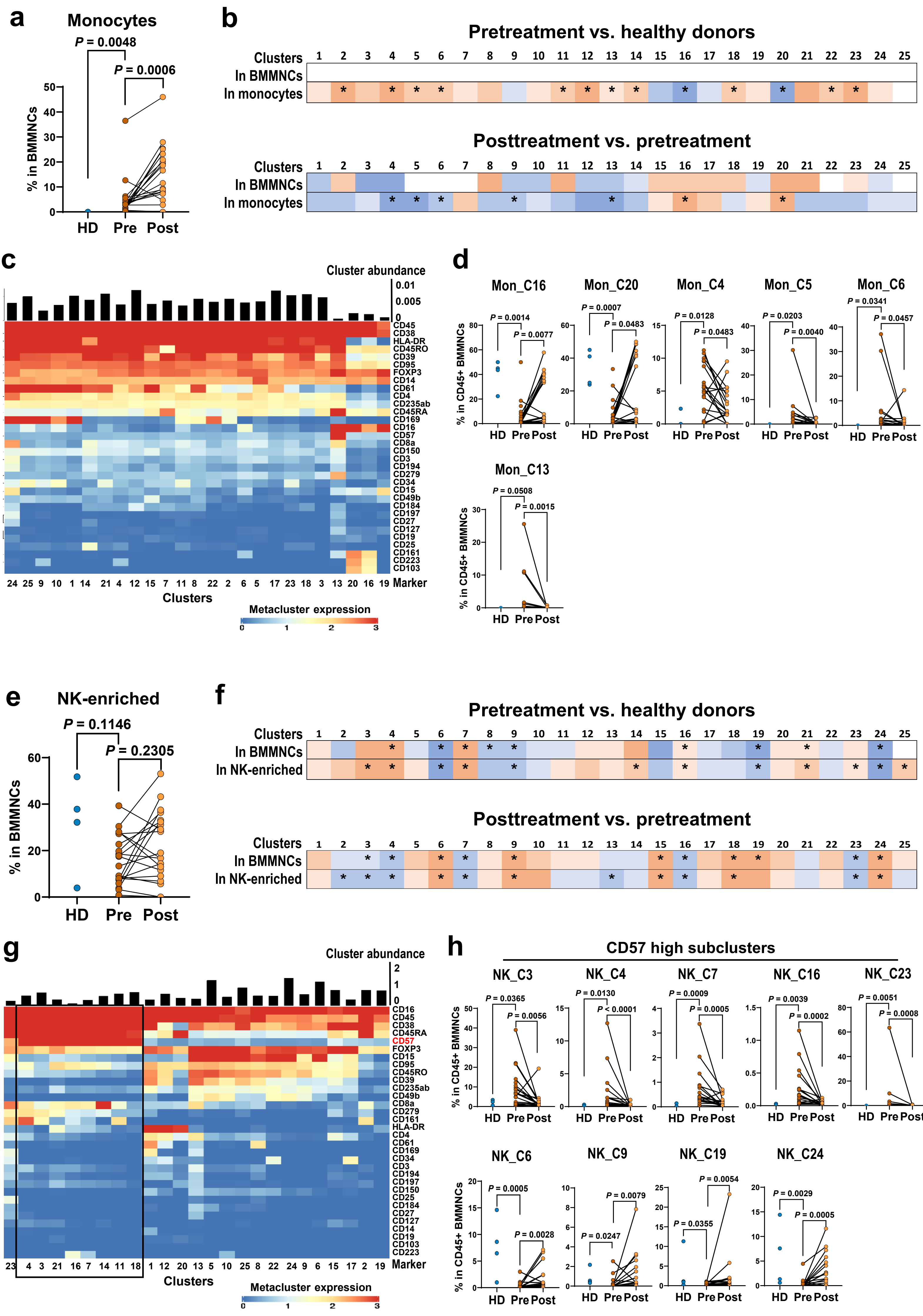
a
Monocytes
P = 0.0048
P = 0.0006
% in BMMNCs
HD Pre Post
b
Pretreatment vs. healthy donors
Clusters
In BMMNCs
In monocytes
Posttreatment vs. pretreatment
c
Cluster abundance
Metacluster expression
Clusters
Marker
d
Mon_C16
Mon_C20
Mon_C4
Mon_C5
Mon_C6
Mon_C13
% in CD45+ BMMNCs
e
NK-enriched
P = 0.1146
P = 0.2305
f
In NK-enriched
g
h
CD57 high subclusters
NK_C3
NK_C4
NK_C7
NK_C16
NK_C23
NK_C6
NK_C9
NK_C19
NK_C24

Supplementary Fig. 8 Phenotyping of monocyte and NK subclusters using CyTOF. a, A scatter plot showing frequency (% in $CD45^+$ BMMNCs) of monocytes of HD (*n* = 4), pretreatment (*n* = 20) and posttreatment samples (*n* = 16). *P* values with the two-sided unpaired and paired Mann-Whitney test. **b**, Heatmaps showing difference of frequency of 25 monocyte cell clusters in pretreatment samples versus HD (top) and in posttreatment versus pretreatment samples (bottom). Red color indicates fold change to be > 1, and blue color indicates fold change to be < 1. Stars present statistical significance ($P < 0.05$; *P* values were calculated with the two-sided unpaired Mann-Whitney test). **c**, Binary clustering of 25 clusters of monocytes based on surface marker expression. A relative expression bar is shown on the bottom. **d**, Scatter plots showing frequency (% in $CD45^+$ BMMNCs) of representative monocyte clusters of HD (*n* = 4), pretreatment (*n* = 20) and posttreatment samples (*n* = 16). *P* values with the two-sided unpaired and paired Mann-Whitney test. **e,** In the CyTOF analysis, NK cells were defined as CD16+ cells in CD3-CD19-CD235AB-CD14- cells due to limitation of antibody panel. It may decrease the purity of NK cells defined here, so we named this population “NK-enriched cells”. A scatter plot showing frequency (% in $CD45^+$ BMMNCs) of NK-enriched cells of HD (*n* = 4), pretreatment (*n* = 20) and posttreatment samples (*n* = 16). *P* values with the two-sided unpaired and paired Mann-Whitney test are shown. Notably, functionally activated NK cells (CD57 high clusters) were overrepresented in SAA patients and decreased after treatment; the total NK-enriched cell population tended to be higher (% in BMMNCs) in SAA and did not change significantly after treatment. **f**, Heatmaps showing difference of frequency of 25 NK cell clusters in pretreatment samples versus HD (top) and in posttreatment versus pretreatment samples (bottom). Color and start legend same as **b**. **g**, Binary clustering of 25 clusters of NK cells based on surface marker expression. **h**, Scatter plots showing frequency (% in $CD45^+$ BMMNCs) of representative NK cell clusters of HD (*n* = 4), pretreatment (*n* = 20) and posttreatment samples (*n* = 16). *P* values with the two-sided unpaired and paired Mann-Whitney test are shown.

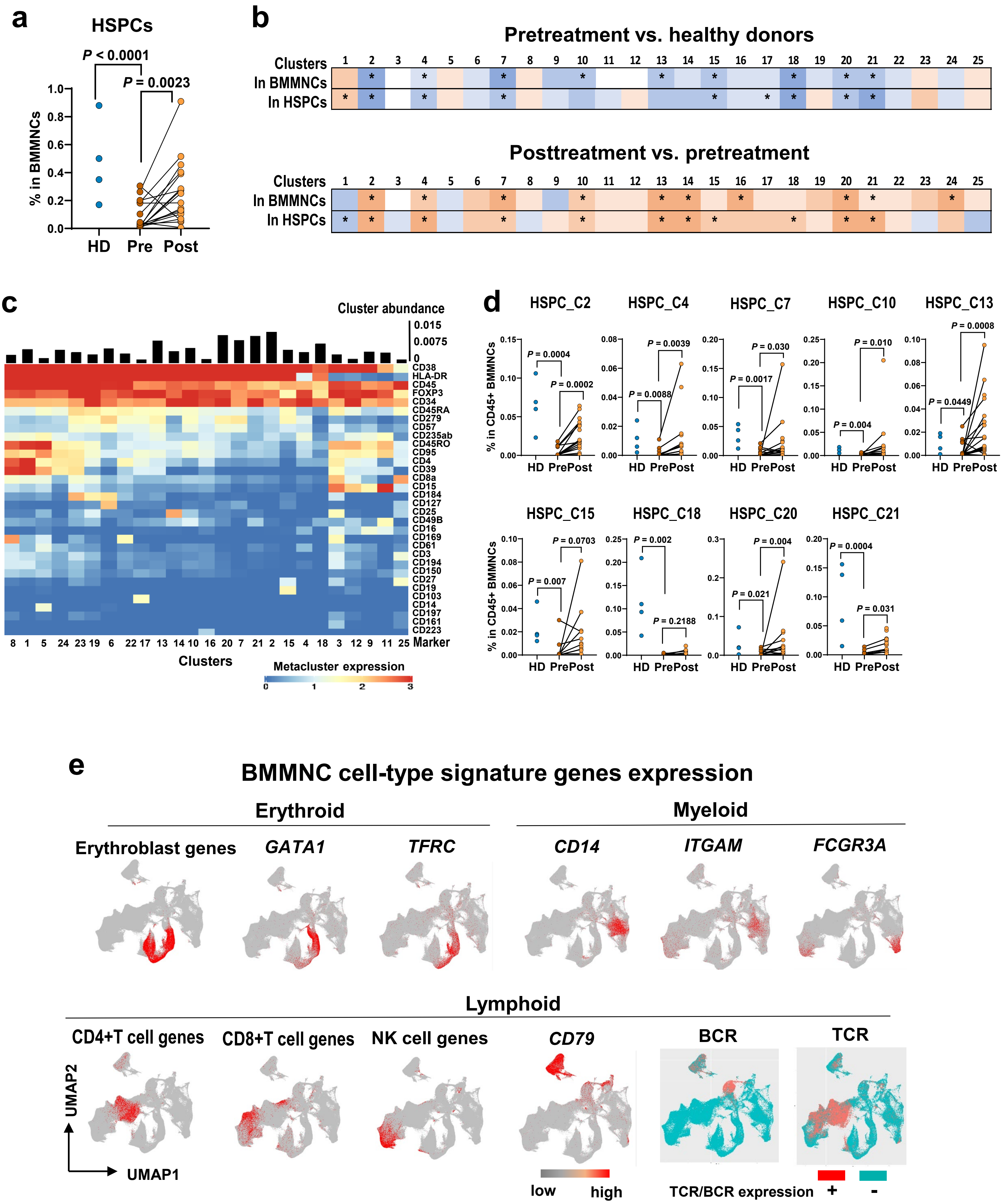
a
HSPCs
P < 0.0001
P = 0.0023
% in BMMNCs
HD
Pre
Post
b
Pretreatment vs. healthy donors
Clusters
In BMMNCs
In HSPCs
Posttreatment vs. pretreatment
c
Cluster abundance
0.015
0.0075
0
CD38
HLA-DR
CD45
FOXP3
CD34
CD45RA
CD279
CD57
CD235ab
CD45RO
CD95
CD4
CD39
CD8a
CD15
CD184
CD127
CD25
CD49B
CD16
CD169
CD61
CD3
CD194
CD150
CD27
CD19
CD103
CD14
CD197
CD161
CD223
Marker
Clusters
Metacluster expression
d
HSPC_C2
HSPC_C4
HSPC_C7
HSPC_C10
HSPC_C13
HSPC_C15
HSPC_C18
HSPC_C20
HSPC_C21
% in CD45+ BMMNCs
P = 0.0004
P = 0.0002
P = 0.0039
P = 0.0088
P = 0.030
P = 0.0017
P = 0.010
P = 0.004
P = 0.0008
P = 0.0449
P = 0.0703
P = 0.007
P = 0.002
P = 0.2188
P = 0.004
P = 0.021
P = 0.0004
P = 0.031
HD PrePost
e
BMMNC cell-type signature genes expression
Erythroid
Myeloid
Erythroblast genes
GATA1
TFRC
CD14
ITGAM
FCGR3A
Lymphoid
CD4+T cell genes
CD8+T cell genes
NK cell genes
CD79
BCR
TCR
UMAP2
UMAP1
low
high
TCR/BCR expression
+
-

Supplementary Fig. 9 Phenotyping of HSPC subclusters using CyTOF. a, A scatter plot showing frequency (% in $CD45^+$ BMMNCs) of HSPCs of healthy controls (*n* = 4), pretreatment samples of SAA patients (*n* = 20) and posttreatment samples (*n* = 16). *P* values with the two-sided unpaired and paired Mann-Whitney test are shown. **b**, Heatmaps showing difference of frequency of 25 monocyte cell clusters in pretreatment samples versus healthy donors (top) and in posttreatment samples versus pretreatment samples (bottom). A red color indicates fold change to be higher than 1, and a blue color indicates fold change to be lower than 1. Stars present statistical significance ($P < 0.05$; *P* values were calculated with the two-sided unpaired Mann-Whitney test). **c**, Binary clustering of 25 clusters of HSPCs based on surface marker expression. A relative expression bar is shown on the bottom. **d**, Scatter plots showing frequency (% in $CD45^+$ BMMNCs) of representative HSPC clusters of healthy controls (*n* = 4), pretreatment samples of SAA patients (*n* = 20) and posttreatment samples (*n* = 16). *P* values with the two-sided unpaired and paired Mann-Whitney test are shown. HSPC, hematopoietic stem and progenitor cell. **e,** Expression of lineage signature genes, cell-type specific genes and TCR/BCR are highlighted in UMAP lots of batch-corrected single-cell gene expression in BMMNCs of all samples at different time points of SAA patients and healthy donors: the same UMAP plot in Fig. 1h.

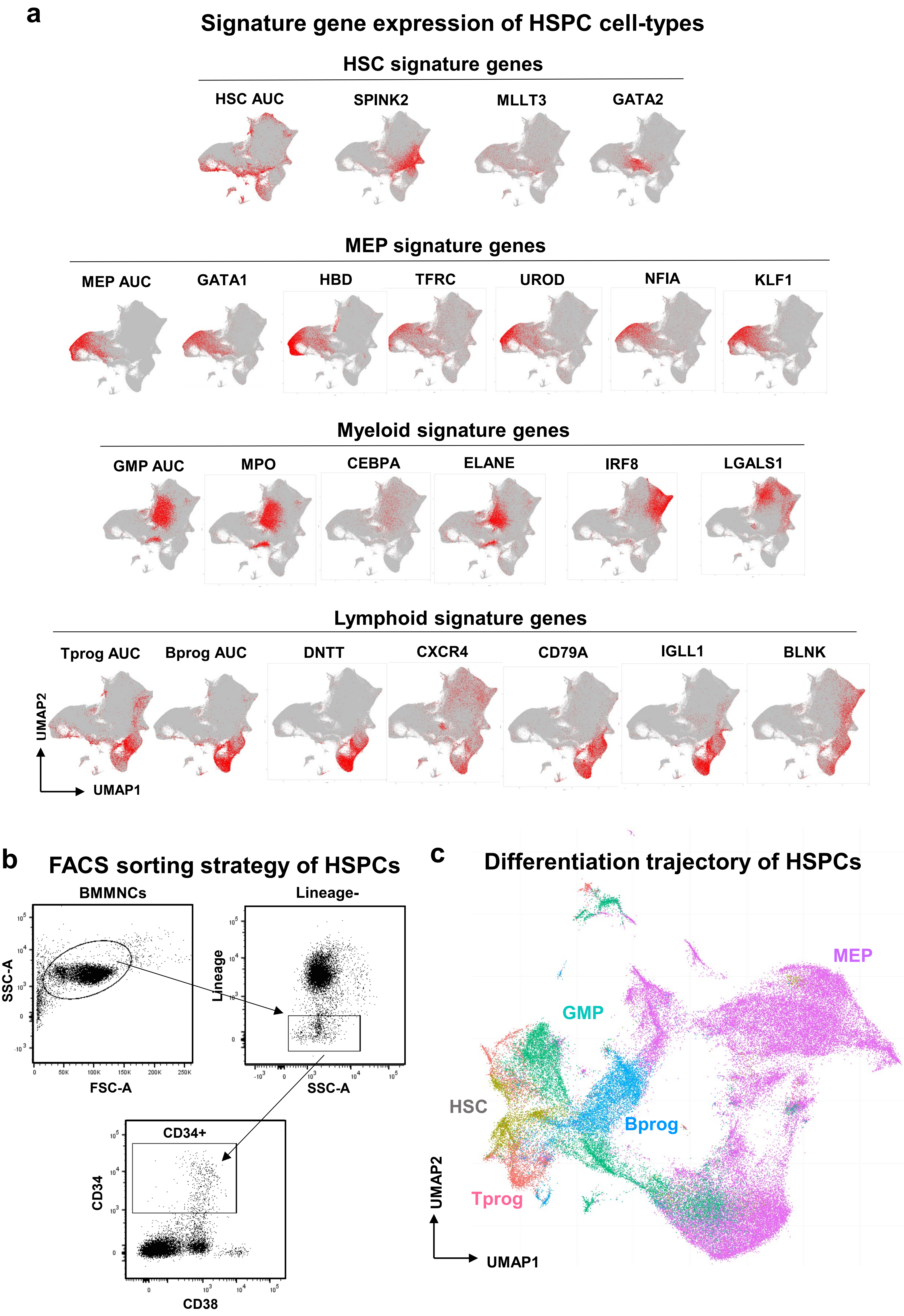
a
Signature gene expression of HSPC cell-types
HSC signature genes
HSC AUC
SPINK2
MLLT3
GATA2
MEP signature genes
MEP AUC
GATA1
HBD
TFRC
UROD
NFIA
KLF1
Myeloid signature genes
GMP AUC
MPO
CEBPA
ELANE
IRF8
LGALS1
Lymphoid signature genes
Tprog AUC
Bprog AUC
DNTT
CXCR4
CD79A
IGLL1
BLNK
UMAP2
UMAP1
b
FACS sorting strategy of HSPCs
BMMNCs
SSC-A
FSC-A
Lineage-
Lineage
SSC-A
CD34+
CD34
CD38
c
Differentiation trajectory of HSPCs
MEP
GMP
HSC
Bprog
Tprog
UMAP2
UMAP1

Supplementary Fig. 10 Resolving heterogeneity of HSPCs using scRNA-seq in SAA. a, Expression of lineage signature genes are highlighted in UMAP lots of batch-corrected single-cell gene expression in HSPCs of all samples at different time points of SAA patients and healthy donors: the same UMAP plot in Fig. 2c. **b,** FACS sorting strategy for Lineage-CD34+ HSPCs from BMMNC samples. FACS data analyses shown in Fig. 2a correspond to Lineage-CD34+ HSPCs. **c,** Reconstruction of the hematopoietic hierarchy pseudotime ordering with Palantir. A color legend is the same as in Fig. 2c.

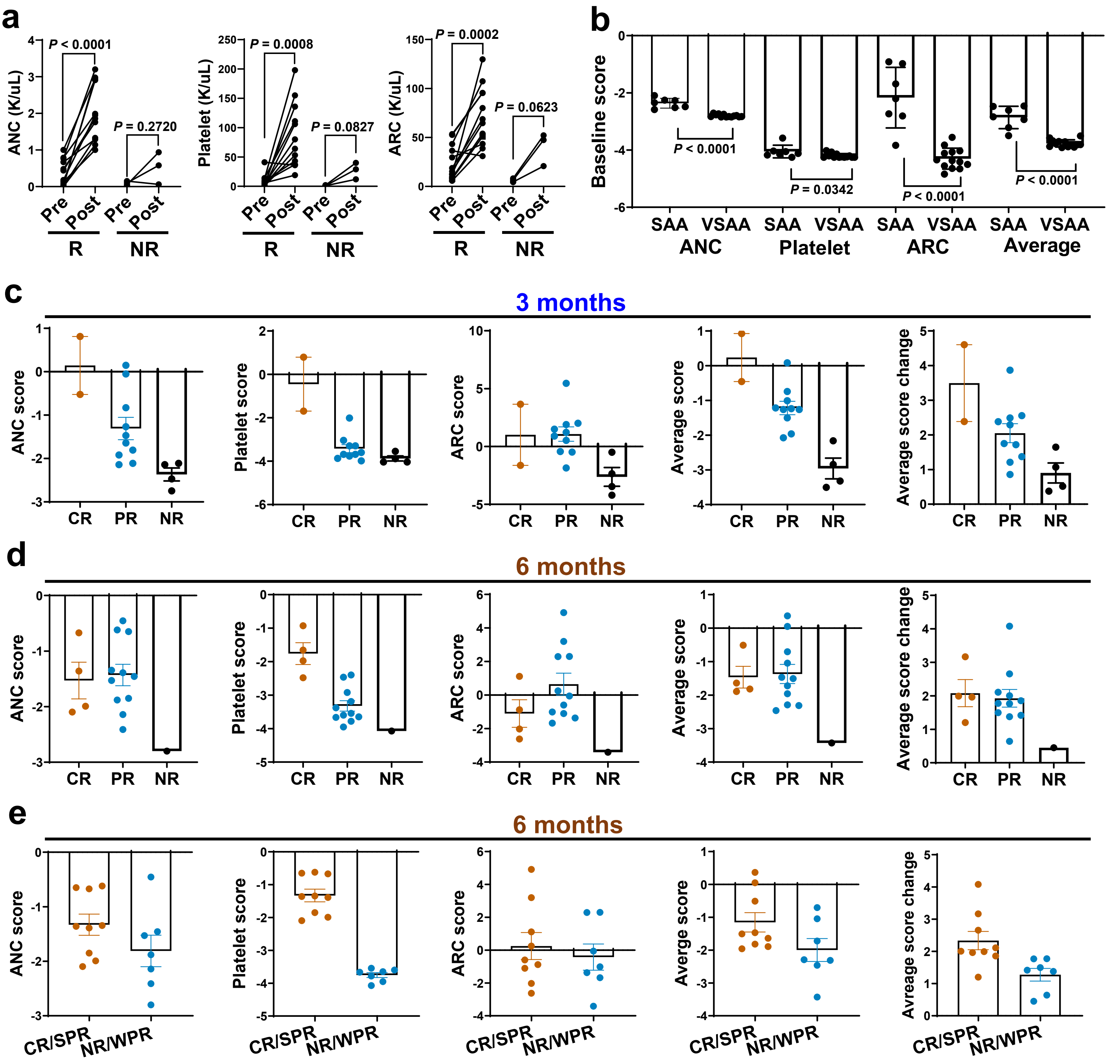

Supplementary Fig. 11 Converted blood count scores reflect a hematopoiesis level and hematopoietic recovery in SAA. a, Scatter plots showing blood counts (ANC, platelet, and ARC) at baseline and after IST in responders and non-responders, respectively, in a total of 20 patients. *P* values with the two-sided paired Mann-Whitney test are shown. **b**, A scatter plot showing blood count scores, including ANC score, platelet score, ARC score and an average blood count score at baseline in SAA and VSAA patients (*n* = 20). These blood count scores were calculated based on blood counts (Methods). Data are presented as mean values ± SEM. *P* values with the two-sided unpaired Mann-Whitney test are shown. **c**, Scatter plots showing blood count scores, including ANC score, platelet score, ARC score, an average blood count score at 3 months and a change of average blood count score at 3 months after IST, in CR, PR and NR patients (*n* = 20). Data are presented as mean values ± SEM. **d**, Scatter plots showing blood count scores, including ANC score, platelet score, ARC score, an average blood count score at 6 months and a change of average blood count score at 6 months after IST, in CR, PR and NR patients (*n* = 20). Data are presented as mean values ± SEM. **e**, Scatter plots showing blood count scores, including ANC score, platelet score, ARC score, an average blood count score at 6 months and a change of average blood count score at 6 months after IST, in CR/SPR and NR/WPR patients (*n* = 20). Data are presented as mean values ± SEM. TCR, T-cell receptor; BCR, B-cell receptor; ANC, absolute neutrophil count; ARC, absolute reticulocyte count; VSAA, very severe aplastic anemia; SPR, strong partial response; WPR, weak partial response.

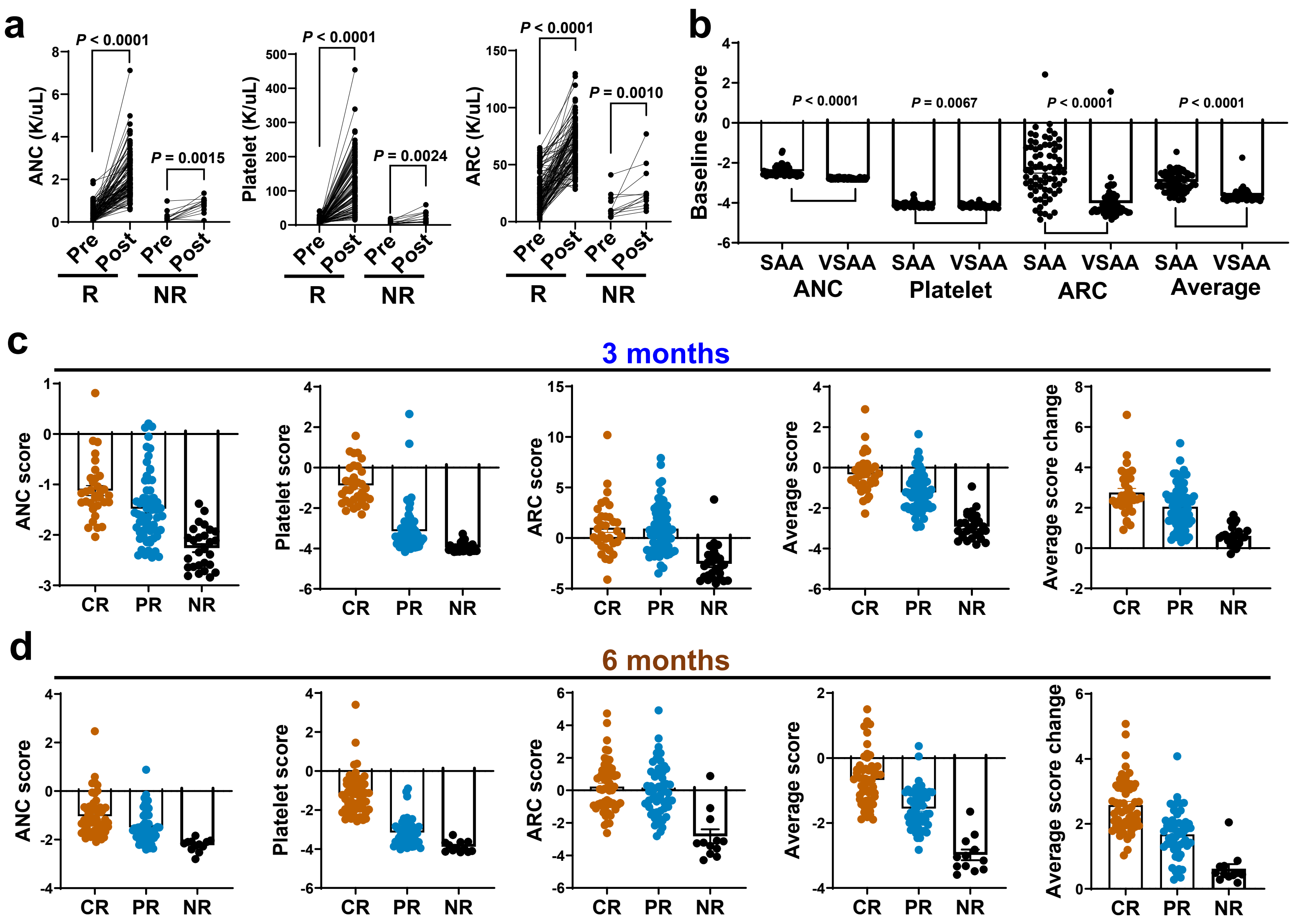

Supplementary Fig. 12 Converted blood count scores reflect a hematopoiesis level and hematopoietic recovery in SAA. a, Scatter plots showing blood counts (ANC, platelet, and ARC) at baseline and after IST in responders and non-responders, respectively, in a total of 137 patients. *P* values with the two-sided paired Mann-Whitney test are shown. **b**, A scatter plot showing blood count scores, including ANC score, platelet score, ARC score and an average blood count score at baseline in SAA and VSAA patients (*n* = 137). These blood count scores were calculated based on blood counts (Methods). Data are presented as mean values ± SEM**.** *P* values with the two-sided unpaired Mann-Whitney test are shown. **c**, Scatter plots showing blood count scores, including ANC score, platelet score, ARC score, an average blood count score at 3 months and a change of average blood count score at 3 months after IST, in CR, PR and NR patients (*n* = 137). Data are presented as mean values ± SEM. **d**, Scatter plots showing blood count scores, including ANC score, platelet score, ARC score, an average blood count score at 6 months and a change of average blood count score at 6 months after IST, in CR, PR and NR patients (*n* = 137). Data are presented as mean values ± SEM**.**

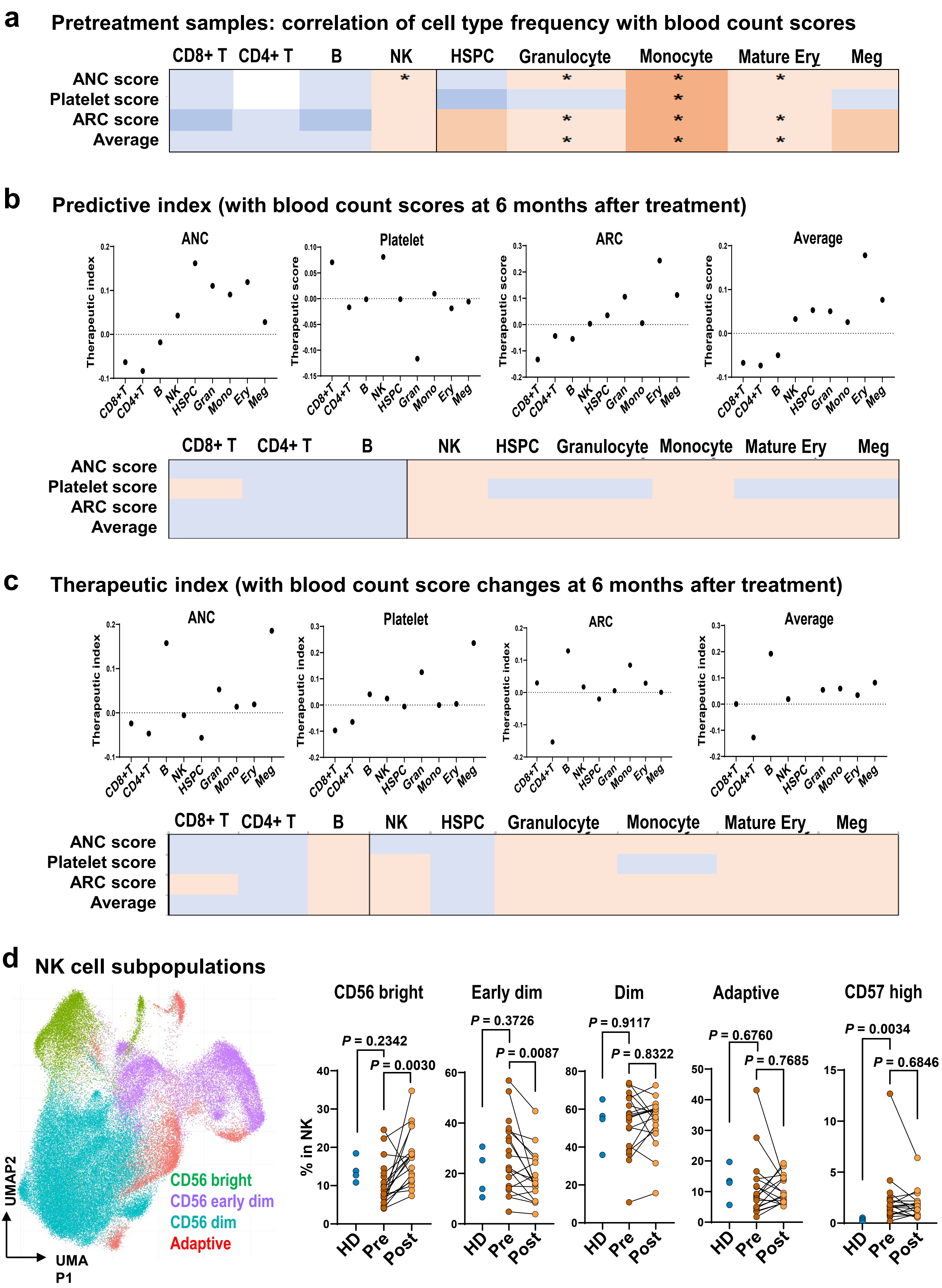
a
Pretreatment samples: correlation of cell type frequency with blood count scores
CD8+ T
CD4+ T
B
NK
HSPC
Granulocyte
Monocyte
Mature Ery
Meg
ANC score
Platelet score
ARC score
Average
b
Predictive index (with blood count scores at 6 months after treatment)
ANC
Platelet
ARC
Average
Therapeutic index
Therapeutic score
CD8+T
CD4+T
B
NK
HSPC
Gran
Mono
Ery
Meg
c
Therapeutic index (with blood count score changes at 6 months after treatment)
ANC
Platelet
ARC
Average
Therapeutic index
d
NK cell subpopulations
CD56 bright
CD56 early dim
CD56 dim
Adaptive
UMAP2
UMAP1
CD56 bright
Early dim
Dim
Adaptive
CD57 high
% in NK
P = 0.2342
P = 0.0030
P = 0.3726
P = 0.0087
P = 0.9117
P = 0.8322
P = 0.6760
P = 0.7685
P = 0.0034
P = 0.6846
HD
Pre
Post

Supplementary Fig. 13 Using predictive and therapeutic indexes to quantify correlation of cell type frequency with blood count scores and blood count score changes. a, A heatmap showing correlation of cell type frequency in pretreatment samples with baseline blood count scores, including ANC, platelet, ARC and an average blood count score. A red color indicates a positive correlation, and a blue color indicates a negative correlation. Stars present statistical significance ($P < 0.05$; P values were calculated with the two-sided unpaired Mann-Whitney test). **b**, Predictive indexes for various cell types indicating correlation of cell type frequency with blood count scores at 6 months after IST. **c**, Therapeutic indexes for various cell types indicating correlation of cell type frequency with blood count score changes at 6 months after IST. **d**, UMAP projection of NK cells, which were assigned to four subtypes: CD56 bright, CD56 early dim, CD56 dim and adaptive NK cells. Scatter plots showing frequency (% in NK) of four subtypes of healthy controls ($n = 4$), pretreatment samples of SAA patients ($n = 20$) and posttreatment samples ($n = 16$). P values with the two-sided unpaired and paired Mann-Whitney test are shown.

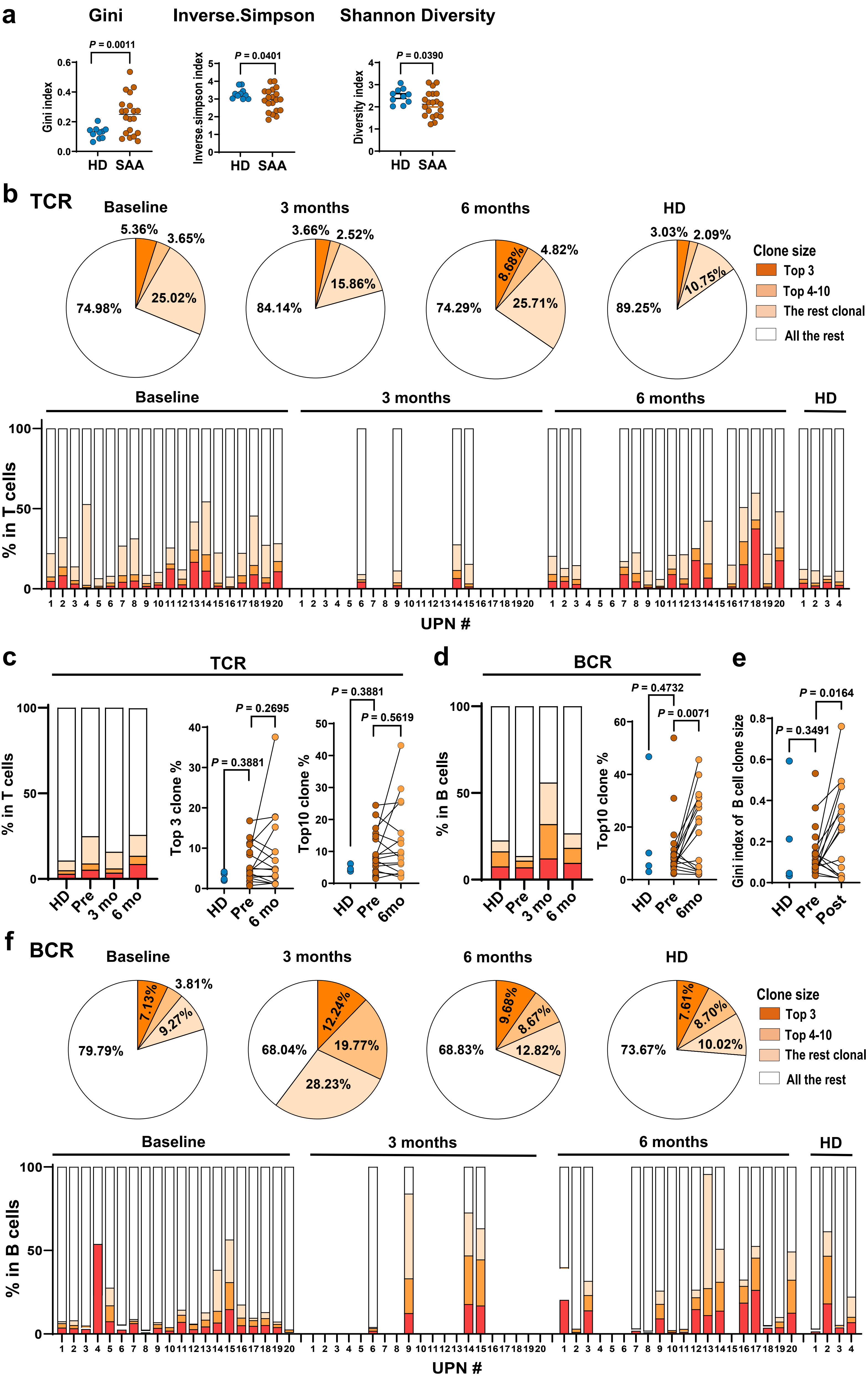
a
Gini
Inverse.Simpson
Shannon Diversity
P = 0.0011
P = 0.0401
P = 0.0390
Gini index
Inverse.simpson index
Diversity index
HD SAA
b TCR
Baseline
3 months
6 months
HD
5.36%
3.65%
25.02%
74.98%
3.66%
2.52%
15.86%
84.14%
8.68%
4.82%
25.71%
74.29%
3.03%
2.09%
10.75%
89.25%
Clone size
Top 3
Top 4-10
The rest clonal
All the rest
% in T cells
UPN #
c TCR
% in T cells
HD Pre 3 mo 6 mo
P = 0.2695
P = 0.3881
Top 3 clone %
HD Pre 6 mo
P = 0.3881
P = 0.5619
Top10 clone %
HD Pre 6mo
d BCR
% in B cells
HD Pre 3 mo 6 mo
P = 0.4732
P = 0.0071
Top10 clone %
HD Pre 6mo
e
P = 0.0164
P = 0.3491
Gini index of B cell clone size
HD Pre Post
f BCR
Baseline
3 months
6 months
HD
7.13%
3.81%
9.27%
79.79%
12.24%
19.77%
28.23%
68.04%
9.68%
8.67%
12.82%
68.83%
7.61%
8.70%
10.02%
73.67%
Clone size
Top 3
Top 4-10
The rest clonal
All the rest
% in B cells
UPN #

Supplementary Fig. 14 Clonal T cell expansion in SAA. **a**, A dot plot showing Gini index, Inverse.Simpson index, and Shannon diversity index of TCR clone size in SAA patients (n = 20) and healthy controls (*n* = 10). *P* values with the two-sided unpaired Mann-Whitney test are shown. **b**, Pie charts on the top summarizing frequency of T cell clones in pretreatment, 3-month, 6-month samples and in health controls. A bar chart at the bottom showing frequency of T cell clones (% in T cells) with clone size top 3, top 4-10, all the rest clones (with at least 2 cells with identical TCR) and cells without clonal TCR in individual samples. **c**, A bar chart comparing frequency of T cell clones in pretreatment, 3-month, 6-month samples and in healthy controls. Scatter plots showing frequency of top 3 (left) and top 10 (right) T cell clones in pretreatment, 6-month samples and in healthy controls. **d**, A bar chart comparing frequency of B cell clones in pretreatment, 3-month, 6-month samples and in healthy controls. Scatter plots showing frequency of top 3 (left) and top 10 (right) B cell clones in pretreatment, 6-month samples and in healthy controls. **e,** A scatter plot showing Gini indexes of BCR clone sizes in pre- (*n* = 20), post-treatment (*n* = 16) samples of SAA patients and healthy controls (*n* = 4). *P* values with the two-sided unpaired and paired Mann-Whitney test are shown. **f,** Pie charts on the top summarizing frequency of B cell clones in pretreatment, 3-month, 6-month samples and in health controls. A bar chart at the bottom showing frequency of B cell clones (% in B cells) with clone size top 3, top 4-10, and the rest clones (with at least 2 cells with identical BCR) and cells without clonal BCR in individual samples.

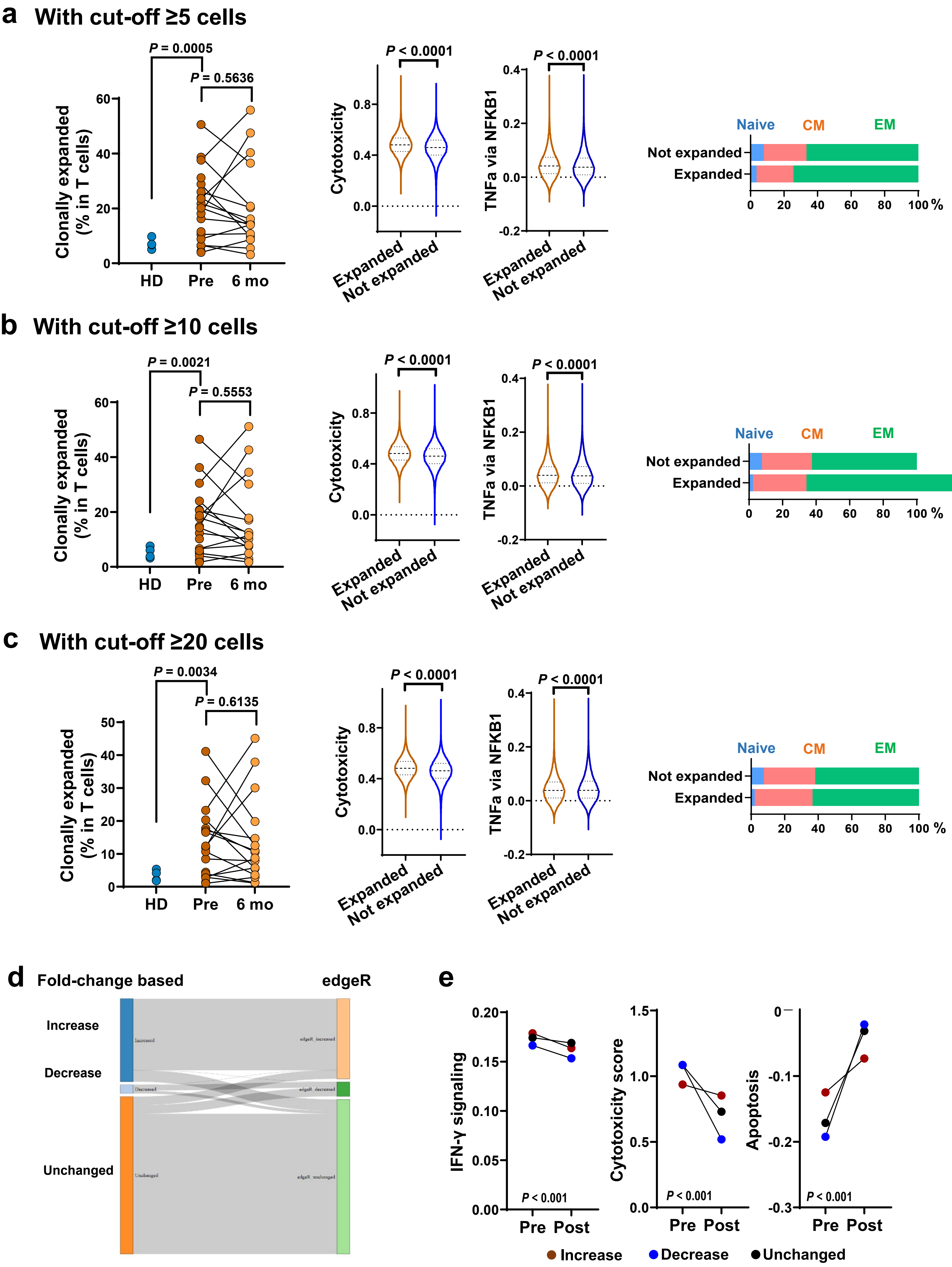
a
With cut-off ≥5 cells
P = 0.0005
P = 0.5636
Clonally expanded (% in T cells)
HD
Pre
6 mo
P < 0.0001
Cytotoxicity
Expanded
Not expanded
TNFa via NFKB1
Naive
CM
EM
Not expanded
Expanded
%
b
With cut-off ≥10 cells
P = 0.0021
P = 0.5553
c
With cut-off ≥20 cells
P = 0.0034
P = 0.6135
d
Fold-change based
edgeR
Increase
Decrease
Unchanged
e
IFN-γ signaling
Cytotoxicity score
Apoptosis
P < 0.001
Pre
Post
Increase
Decrease
Unchanged

Supplementary Fig. 15 Consistency when using different cut-offs for definition of T cell clonal expansion and clone size dynamics. For statistical determination in analysis with an arbitrary (but widely-used) criteria for clone definition (clonally expanded T cells were defined as when there were > 2 cells with identical TCR), same analyses as shown in Fig. 4a and 4c were done with definition of expanded clones as ≥ 5 cells (**a**), ≥ 10 cells (**b**), and ≥ 20 cells (**c**) having identical TCR. Left panel, a dot plot showing frequency of total clonal expanded T cells in pre- (*n* = 20), post-treatment (*n* = 16) samples of SAA patients and healthy controls (*n* = 4). *P* values with the two-sided unpaired and paired Mann-Whitney test are shown. Middle panel, expression of T cytotoxicity genes and TNF-α via NFκB signaling genes were plotted for clonally expanded T cells and T cells not expanded. *P* values with the two-sided unpaired and paired Mann-Whitney test were shown. Right panel, bar chart showing percentage of clonal expanded and nonexpanded T cells as naïve, CM, and EM. Among clones with at least 20 cells (for feasibility of comparison of gene expression), except for defining clonal dynamics based on fold change, edgeR was used to identify clones with an increase, decrease or unchanged dynamics pattern. **d**, A Sankey plot showing overall overlapping results of defining clonal size dynamics using fold change approach and edgeR. **e**, Same analysis as shown in Figure 4d, expression dynamics of IFN-γ signaling, cytotoxicity and cell apoptosis genes of increased, decreased and stable clones (defined by edgeR) after treatment.

a

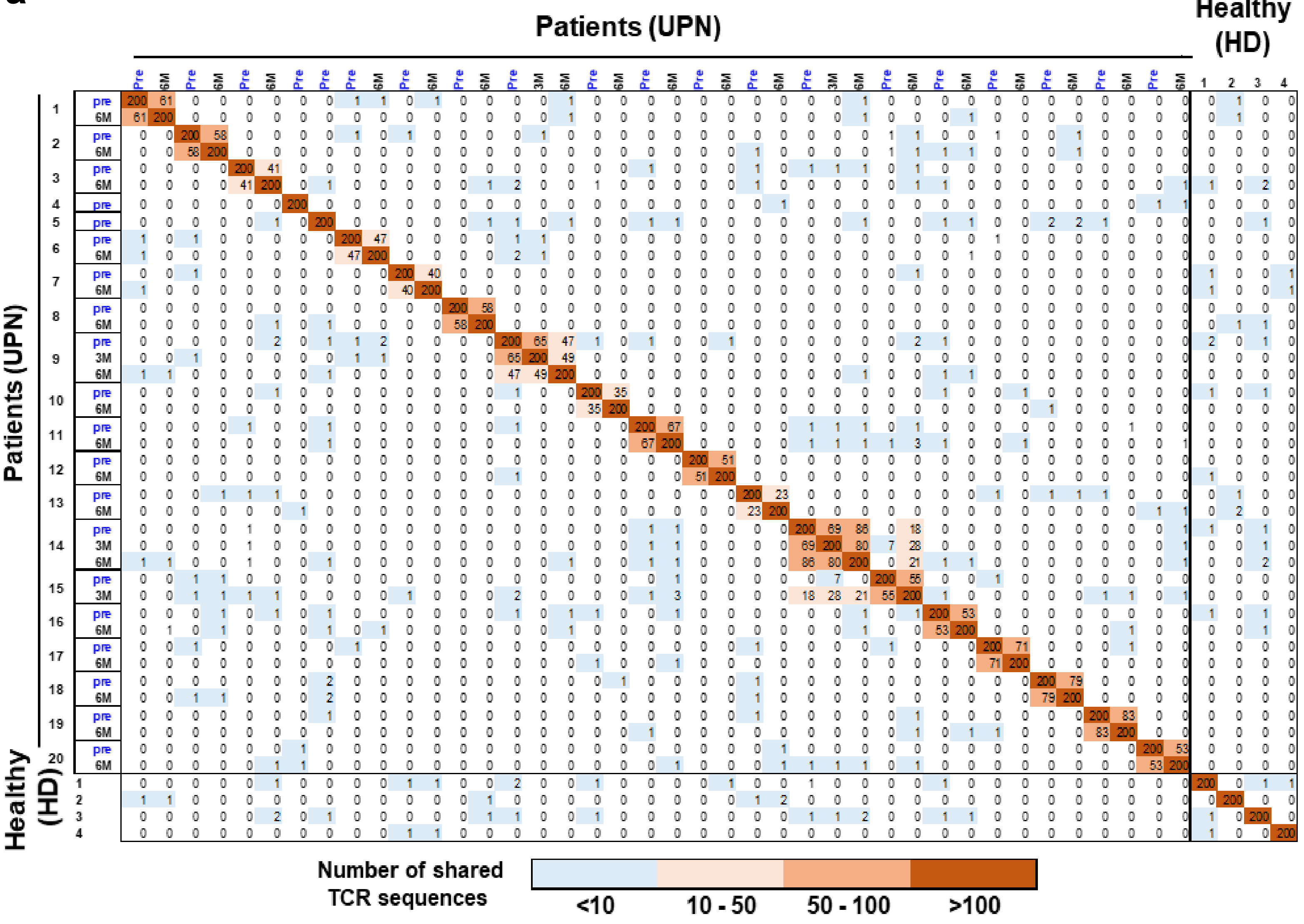
TCR clones with identical CDR3
Patients (UPN)
Healthy (HD)
Patients (UPN)
Healthy (HD)
Number of shared TCR sequences
<10
10 - 50
50 - 100
>100

b

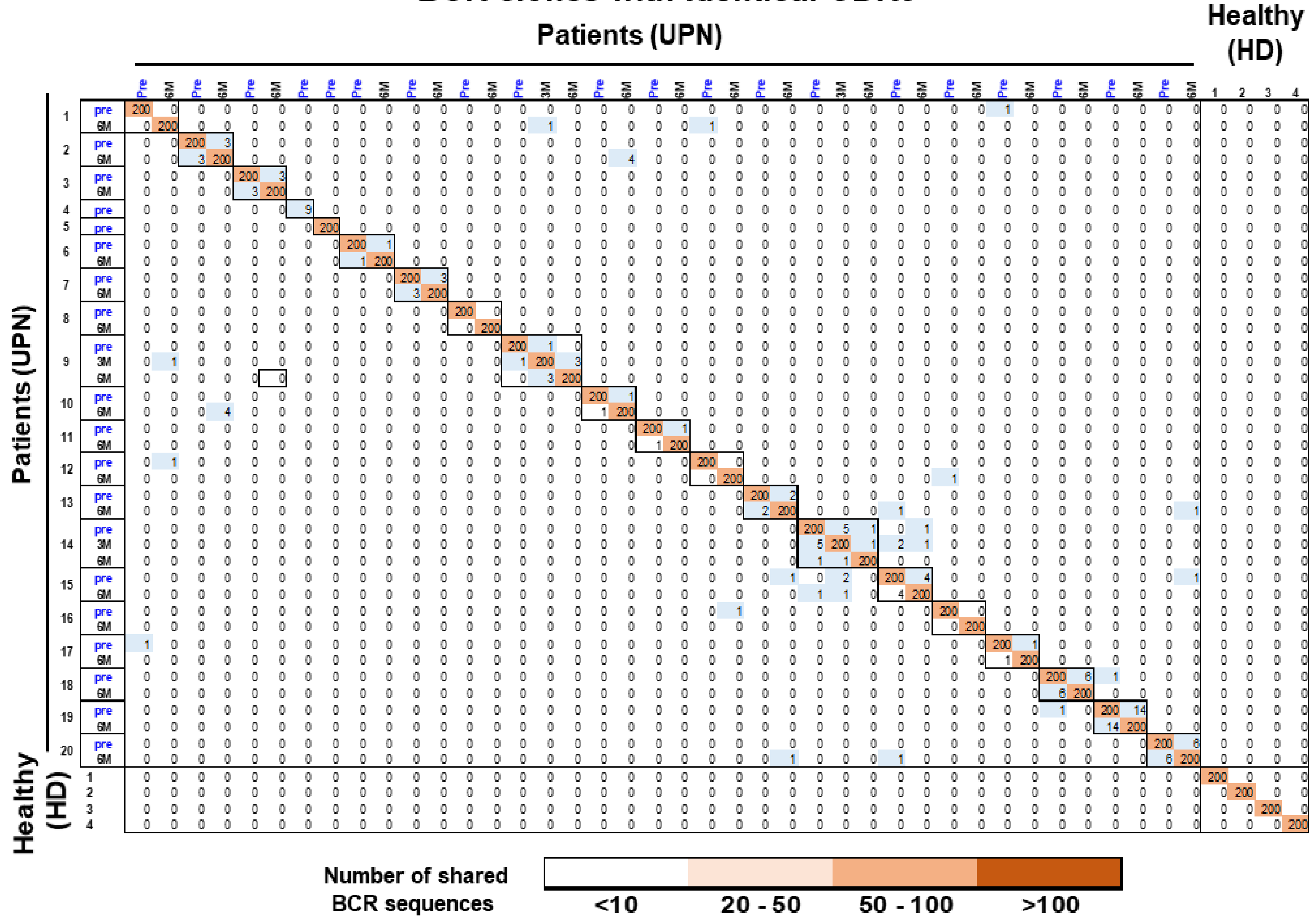
BCR clones with identical CDR3
Patients (UPN)
Healthy (HD)
Patients (UPN)
Healthy (HD)
Number of shared BCR sequences
<10
20 - 50
50 - 100
>100

Supplementary Fig. 16 Lack of common TCR and BCR clonotypes in SAA patients. a, A heatmap plot showing the number of common TCR clones in pre- and posttreatment (3 and/or 6 months) samples of SAA patients (*n* = 20) and healthy controls (*n* = 4) among top 500 TCR clones. Both x-and y-axes represent samples of patients and healthy donors. Paired samples of the same SAA patients were adjacent. Numbers indicate counts of identical TCR clones shared among samples. A color scheme ranging from dark orange to dark blue represents the number of shared CDR sequences from high to low. In general, there was lack of common TCR usage in SAA, and few common TCR clones in healthy individuals or SAA patients. **b**, A heatmap plot showing the number of common BCR clones in pre- and posttreatment samples of SAA patients (*n* = 20) and healthy controls (*n* = 4) among top 500 BCR clones. Both x-and y-axes represent samples of patients and healthy donors. Paired samples of the same SAA patient were adjacent. Numbers indicate counts of identical BCR clones shared among samples. A color scheme ranging from dark orange to dark blue represents the number of shared CDR sequences from high to low. In general, there was lack of common BCR usage in SAA, and few common BCR clones in healthy individuals or SAA patients. CDR, complementarity determining region; HD, healthy donor; UPN, unique patient number; 3M, 3 months; 6M, 6 months.

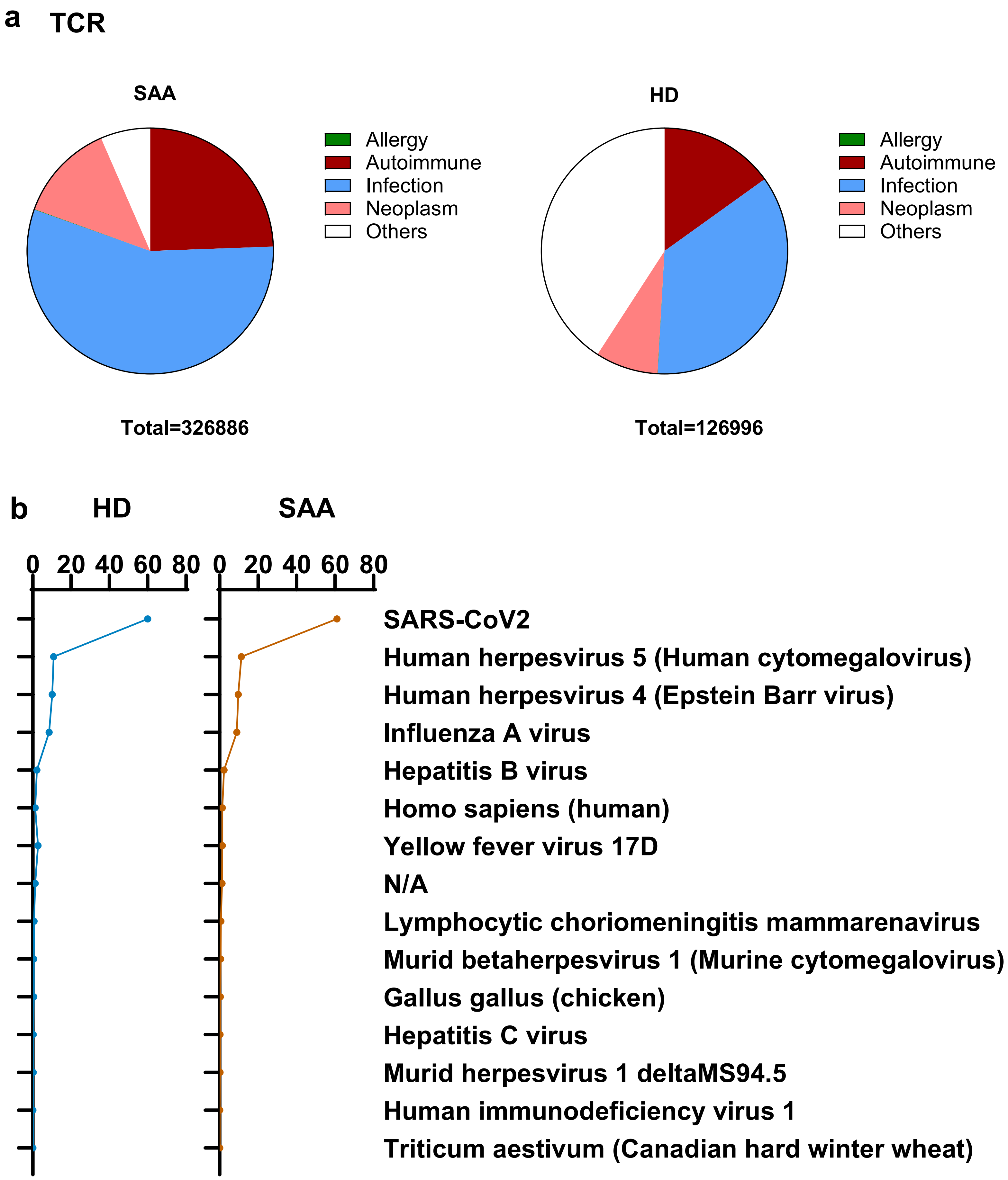

***N/A, not assigned to any virus in IEDB database.**

Supplementary Fig. 17 Interpretation of TCR sequences based on IEDB database. TCR sequences identified in SAA patients and healthy donors were compared to disease-specific or pathogen-specific TCR sequences in IEDB database. **a**, Pie charts showing TCR sequences were grouped to be related with allergy, autoimmune infectious, neoplasm diseases and others. **b**, Plot showing TCR sequences (frequency >0.1%) annotated to be specific to pathogens.

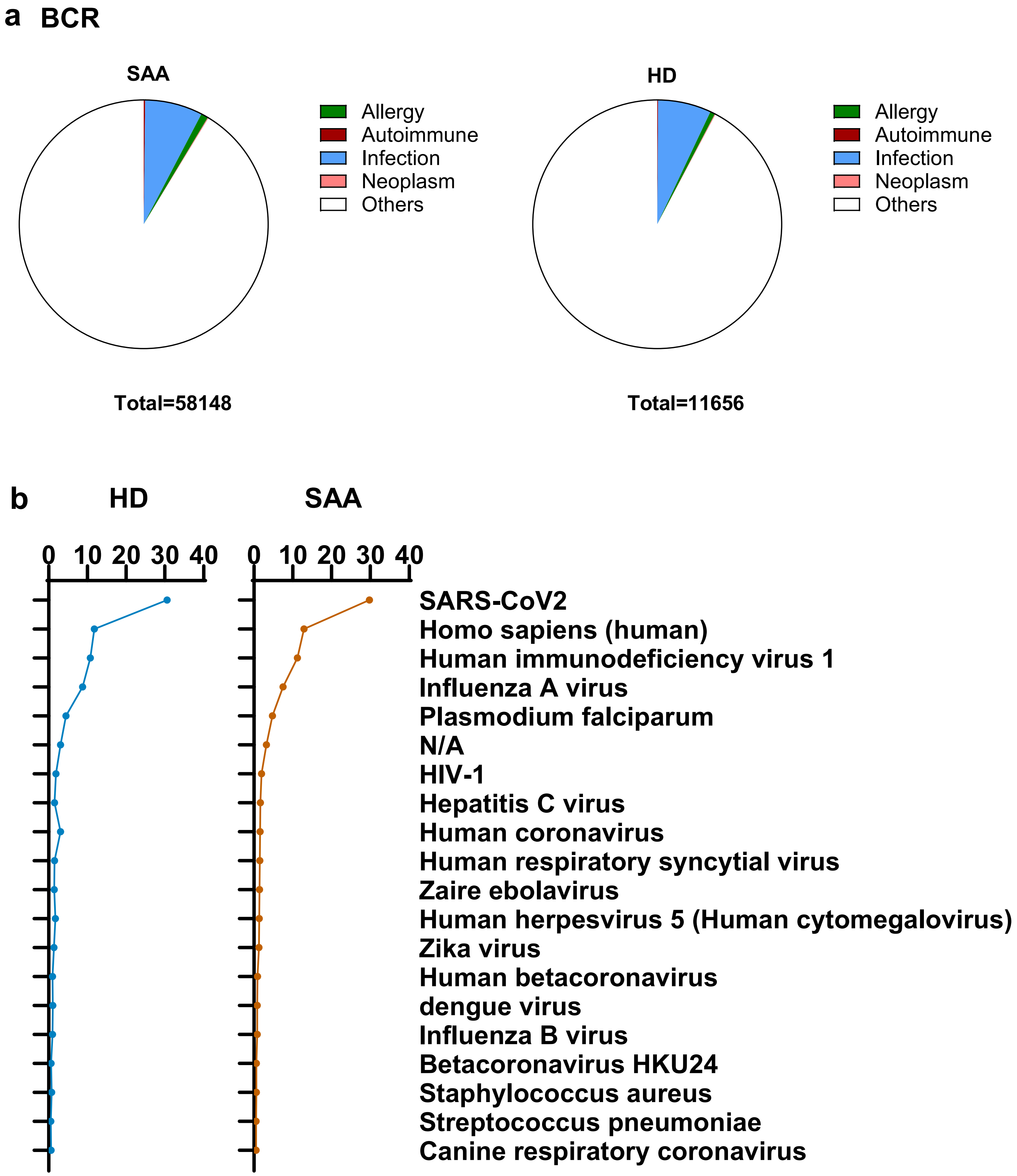

***N/A, not assigned to any virus in IEDB database.**

Supplementary Fig. 18 Interpretation of BCR sequences based on IEDB database. BCR sequences identified in SAA patients and healthy donors were compared to disease-specific or pathogen-specific BCR sequences in IEDB database. **a**, Pie charts showing BCR sequences were grouped to be related with allergy, autoimmune infectious, neoplasm diseases and others. **b**, Plot showing BCR sequences (frequency >0.5%) annotated to be specific to pathogens.

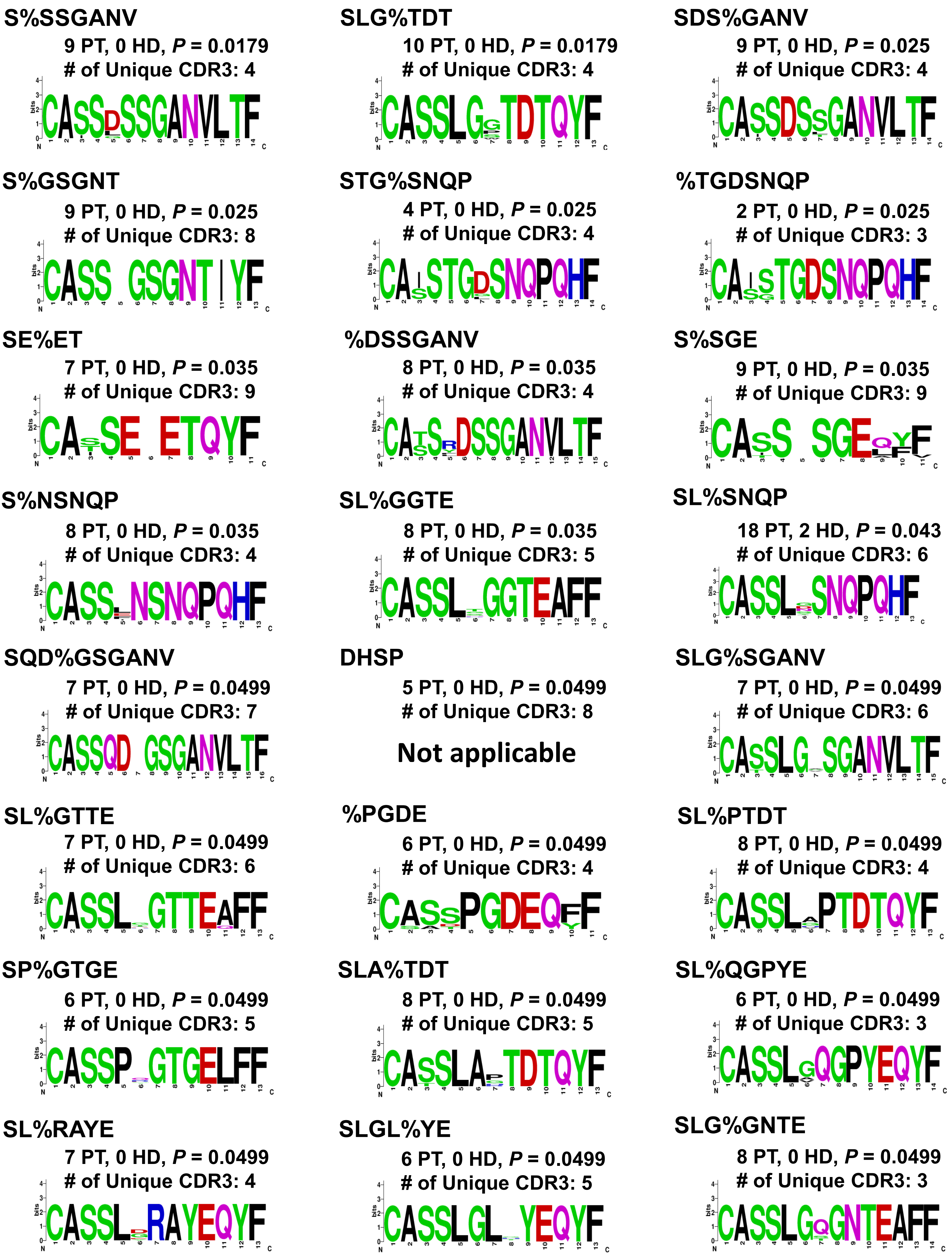

Supplementary Fig. 19 SAA-specific TCR specificity groups. Sequences and corresponding weblogs of 24 TCR specificity groups with different CDRs. In each panel, it indicates a sequence of this TCR specific group, the presence of this TCR specificity group in the number of SAA patients and healthy controls, a *P* value of difference of frequency, and the number of unique CDR3.

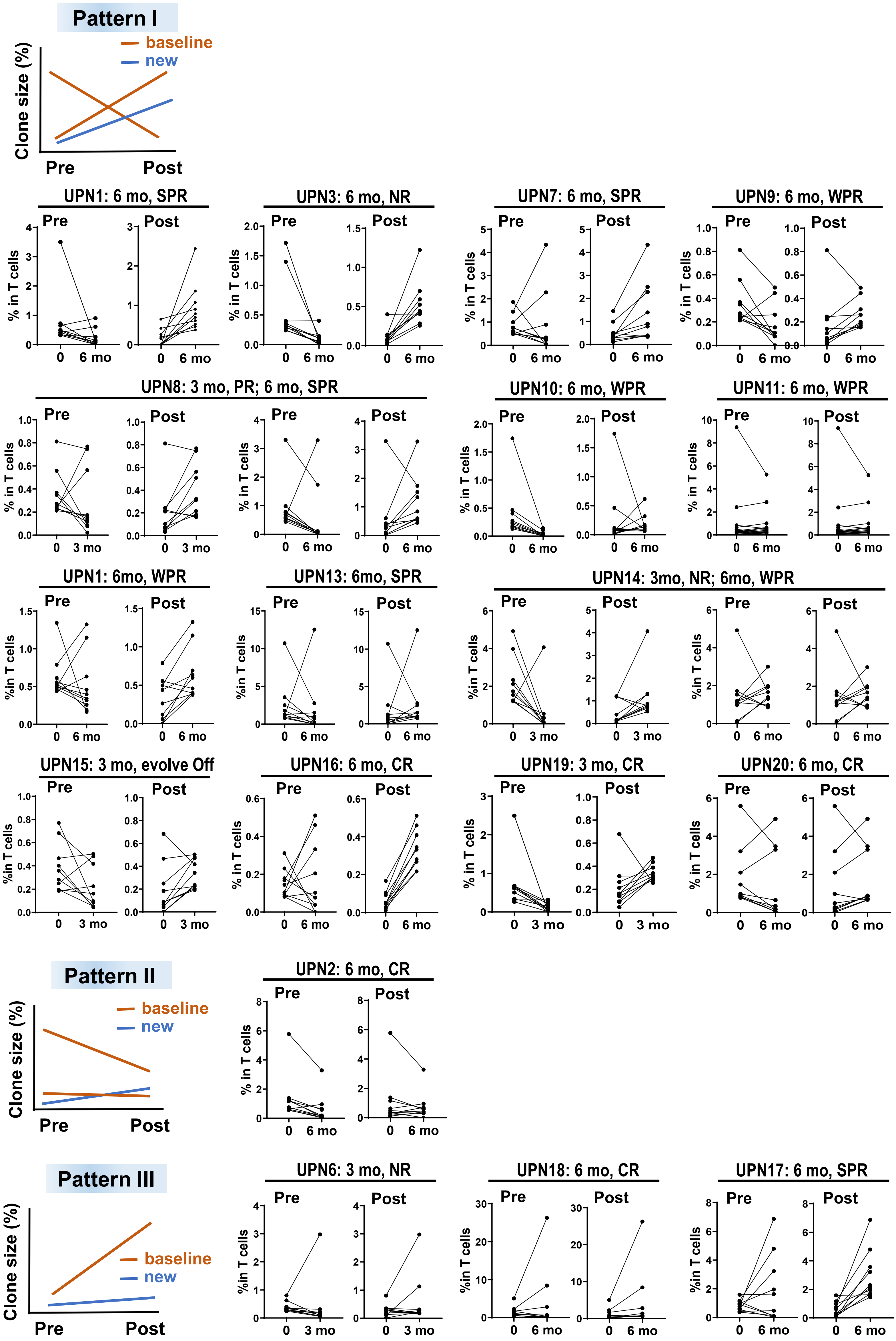
Pattern I
baseline
new
Clone size (%)
Pre
Post
UPN1: 6 mo, SPR
UPN3: 6 mo, NR
UPN7: 6 mo, SPR
UPN9: 6 mo, WPR
UPN8: 3 mo, PR; 6 mo, SPR
UPN10: 6 mo, WPR
UPN11: 6 mo, WPR
UPN1: 6mo, WPR
UPN13: 6mo, SPR
UPN14: 3mo, NR; 6mo, WPR
UPN15: 3 mo, evolve Off
UPN16: 6 mo, CR
UPN19: 3 mo, CR
UPN20: 6 mo, CR
% in T cells
%in T cells
0
3 mo
6 mo
Pattern II
baseline
new
Clone size (%)
Pre
Post
UPN2: 6 mo, CR
Pattern III
baseline
new
Clone size (%)
Pre
Post
UPN6: 3 mo, NR
UPN18: 6 mo, CR
UPN17: 6 mo, SPR

Supplementary Fig. 20 Three patterns of clonal kinetics of SAA patients pre- and posttreatment. For each patient, dynamics of their clones were grouped into three patterns. Pattern 1, many novel clones after treatment and variable dynamics of pre-existing clones (up or down, UPN1, 3, 7, 8, 9, 10, 11, 12, 15, 16, 17 and 19); pattern 2, decrease in pre-existing clones with few novel clones (UPN2); pattern 3, increase in preexisting clones with few novel clones (UPNs 6 and 18). Despite complexity, there was no correlation of clonal dynamics with response to treatment. Each panel includes a diagram illustrating a pattern of clonal kinetics. For each individual, a scatter plot on the left shows top ten clones pretreatment and a plot on the right shows top ten clones posttreatment. UPN, time point, and response at that time point are indicated on top.

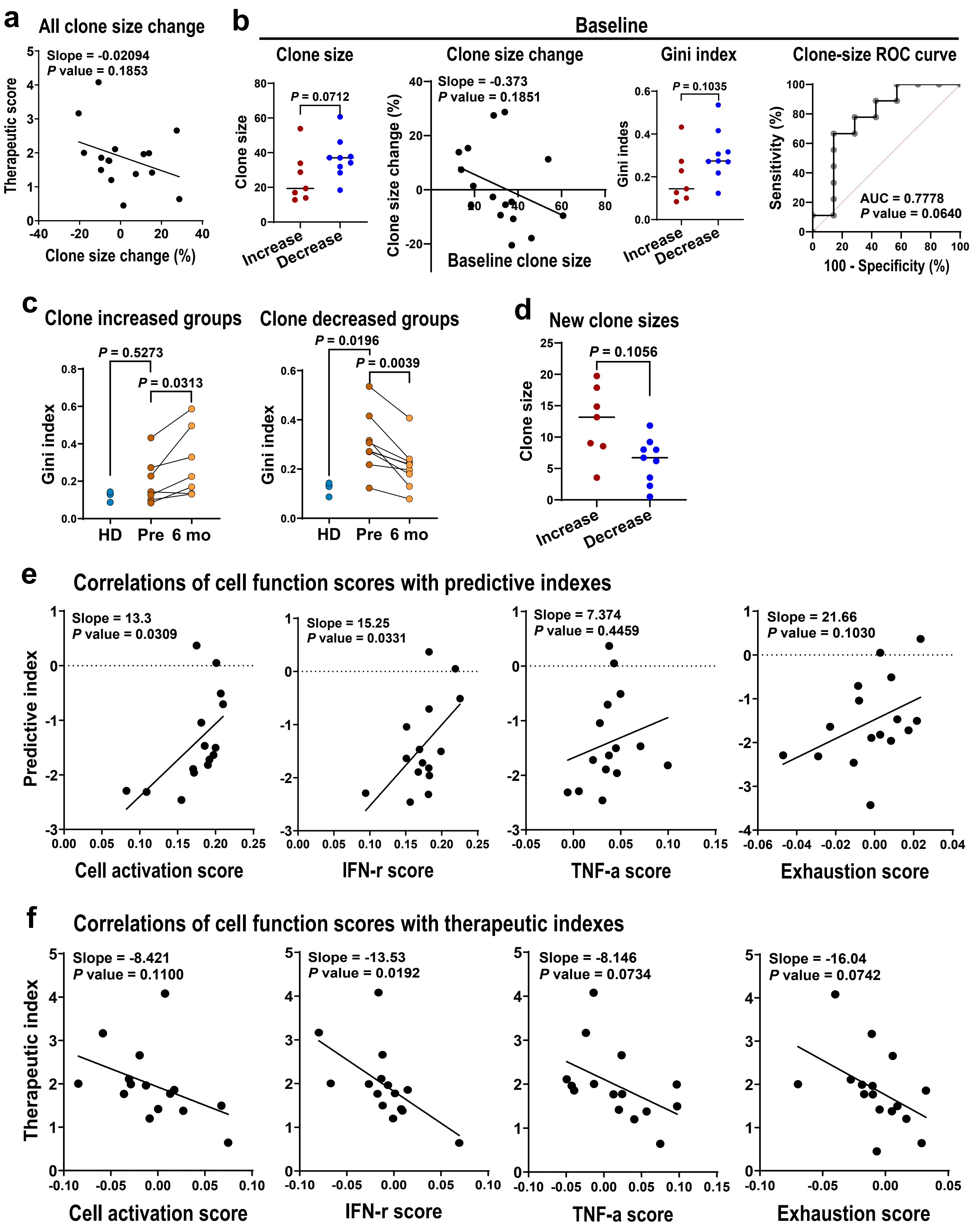

Supplementary Fig. 21 T cell clonal expansion dynamics associates with hematopoietic recovery. a, Correlation of changes of all clone sizes with therapeutic indexes was analyzed. *P* values and slope with the Pearson correlation test are shown. **b**, Clone sizes at baseline were compared in patients who had increased and decreased clone sizes after IST. *P* values with the two-sided unpaired and paired Mann-Whitney test are shown. Correlation of clone sizes at baseline with the changes of clone sizes after IST was analyzed. *P* values and slope with the Pearson correlation test are shown. Gini indexes at baseline were compared in patients who had increased and decreased clone sizes after IST. *P* values with the

two-sided unpaired Mann-Whitney test are shown. A ROC curve of clone sizes at baseline to predict clone size increase or decrease after treatment. AUC and *P* values are shown. **c**, Scatter plots showing Gini indexes of TCR clone sizes in healthy controls (n = 4), pre-, and posttreatment samples of patients who had increased (n = 7) and decrease (n = 9) clone sizes after IST. *P* values with the two-sided unpaired and paired Mann-Whitney test are shown. **d**, Novel clone sizes were compared in patients who had increased and decreased clone sizes after IST. *P* values with the two-sided unpaired Mann-Whitney test are shown. **e**, Correlations of cell function scores (including cell activation, IFN-g signaling, TNF-a signaling and exhaustion) with predictive indexes were analyzed. *P* values and slope with the Pearson correlation test are shown. **f**, Correlations of cell function scores (including cell activation, IFN-g signaling, TNF-a signaling and exhaustion) with therapeutic indexes were analyzed. *P* values and slope with the Pearson correlation test are shown.

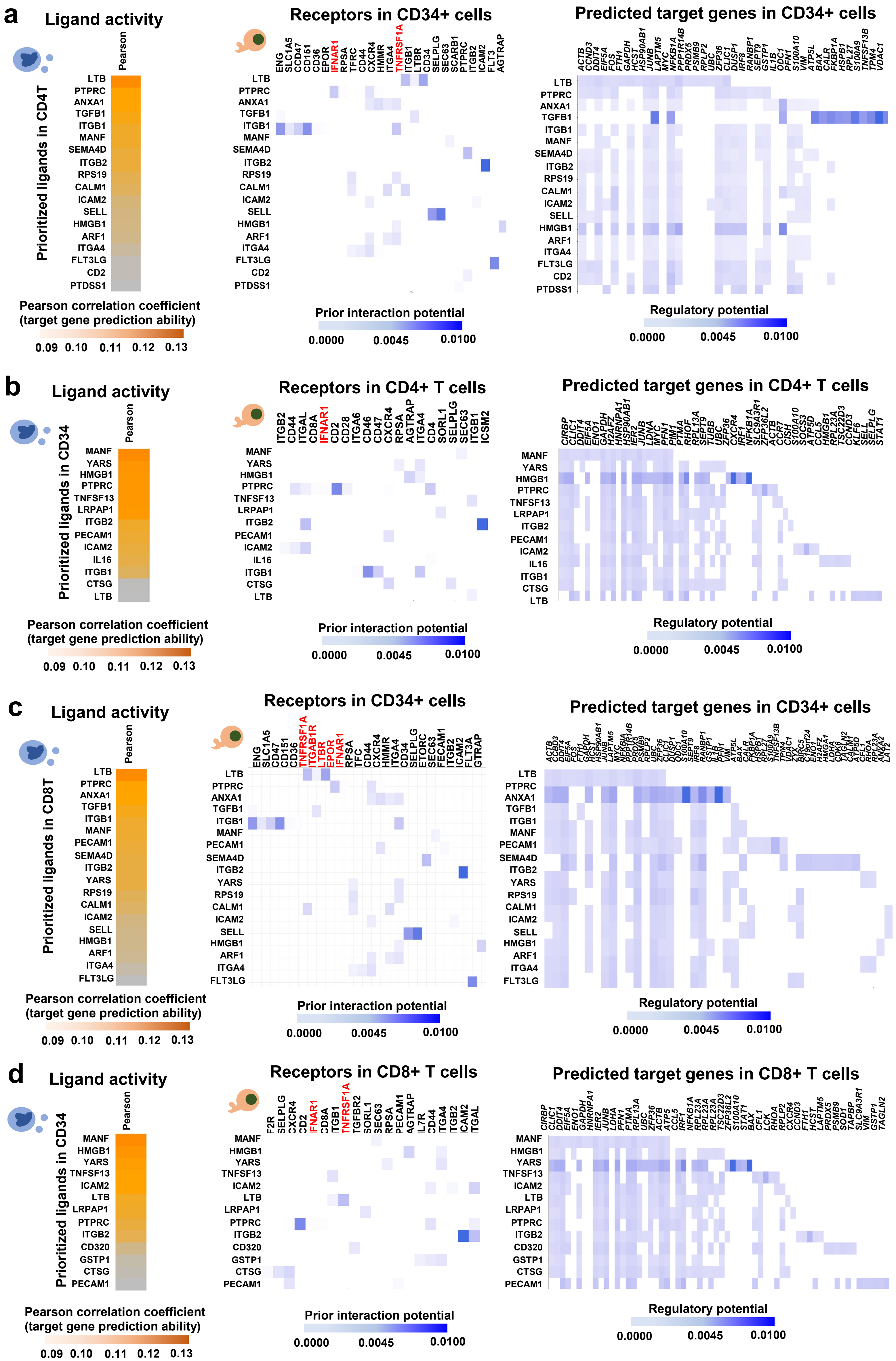
a
Ligand activity
Pearson
Prioritized ligands in CD4T
LTB
PTPRC
ANXA1
TGFB1
ITGB1
MANF
SEMA4D
ITGB2
RPS19
CALM1
ICAM2
SELL
HMGB1
ARF1
ITGA4
FLT3LG
CD2
PTDSS1
Pearson correlation coefficient
(target gene prediction ability)
0.09 0.10 0.11 0.12 0.13
Receptors in CD34+ cells
ENG SLC1A5 CCD47 CD151 CD36 EPOR IFNAR1 RPSA TFRC CD44 CXCR4 HMMR ITGA4 TNFRSF1A ITGB1 LTBR CD34 SELPLG SEC63 SCARB1 PTPRC ITGB2 ICAM2 FLT3 AGTRAP
Prior interaction potential
0.0000 0.0045 0.0100
Predicted target genes in CD34+ cells
Regulatory potential
0.0000 0.0045 0.0100
b
Ligand activity
Pearson
Prioritized ligands in CD34
MANF
YARS
HMGB1
PTPRC
TNFSF13
LRPAP1
ITGB2
PECAM1
ICAM2
IL16
ITGB1
CTSG
LTB
Pearson correlation coefficient
(target gene prediction ability)
0.09 0.10 0.11 0.12 0.13
Receptors in CD4+ T cells
ITGB2 CD44 ITGAL CD8A IFNAR1 CD2 CD28 ITGA6 CD46 CD47 CXCR4 RPSA AGTRAP ITGA4 CD4 SORL1 SELPLG SEC63 ITGB1 ICSM2
Prior interaction potential
0.0000 0.0045 0.0100
Predicted target genes in CD4+ T cells
Regulatory potential
0.0000 0.0045 0.0100
c
Ligand activity
Pearson
Prioritized ligands in CD8T
LTB
PTPRC
ANXA1
TGFB1
ITGB1
MANF
PECAM1
SEMA4D
ITGB2
YARS
RPS19
CALM1
ICAM2
SELL
HMGB1
ARF1
ITGA4
FLT3LG
Pearson correlation coefficient
(target gene prediction ability)
0.09 0.10 0.11 0.12 0.13
Receptors in CD34+ cells
ENG SLC1A5 CD47 CD151 CD36 TNFRSF1A ITGAB1R LTBR EPOR IFNAR1 RPSA TFC CD44 CXCR4 HMMR ITGA4 CD34 SELPLG ETORC SEC63 PECAM1 ITGB2 ICAM2 FLT3A GTRAP
Prior interaction potential
0.0000 0.0045 0.0100
Predicted target genes in CD34+ cells
Regulatory potential
0.0000 0.0045 0.0100
d
Ligand activity
Pearson
Prioritized ligands in CD34
MANF
HMGB1
YARS
TNFSF13
ICAM2
LTB
LRPAP1
PTPRC
ITGB2
CD320
GSTP1
CTSG
PECAM1
Pearson correlation coefficient
(target gene prediction ability)
0.09 0.10 0.11 0.12 0.13
Receptors in CD8+ T cells
F2R SELPLG CXCR4 CD2 IFNAR1 CD8A ITGB1 TNFRSF1A TGFBR2 SORL1 SEC63 RPSA PECAM1 AGTRAP IL7R CD44 ITGA4 ITGB2 ICAM2 ITGAL
Prior interaction potential
0.0000 0.0045 0.0100
Predicted target genes in CD8+ T cells
Regulatory potential
0.0000 0.0045 0.0100

Supplementary Fig. 22 Enhanced cell-cell interactions in SAA. Cell-cell interactions were defined by NicheNetr.[51] **a**, Ligands expressed by $CD4^+$ T cells were ranked by likelihood that ligands would affect gene expression changes in $CD34^+$ HSPCs. Receptors expressed on $CD34^+$ HSPCs were selected based on their known potential to interact with prioritized ligands. Finally, target genes were selected based on their differential expression in $CD34^+$ HSPCs and their potential to be regulated by ligand-receptor interactions identified between $CD4^+$ T cells and $CD34^+$ HSPCs. **b**, Ligands expressed by $CD34^+$ HSPCs were ranked by likelihood that ligands would affect gene expression changes in $CD4^+$ T cells. Receptors expressed on $CD4^+$ T were selected based on their known potential to interact with prioritized ligands. Finally, target genes were selected based on their differential expression in $CD4^+$ T and their potential to be regulated by ligand-receptor interactions identified between $CD34^+$ HSPCs and $CD4^+$ T cells. **c**, Ligands expressed by $CD8^+$ T cells were ranked by likelihood that ligands would affect gene expression changes in $CD34^+$ HSPCs. Receptors expressed on $CD34^+$ HSPCs were selected based on their known potential to interact with prioritized ligands. Finally, target genes were selected based on their differential expression in $CD34^+$ HSPCs and their potential to be regulated by ligand-receptor interactions identified between $CD8^+$ T cells and $CD34^+$ HSPCs. Those downstream genes are involved in HSPC differentiation, cell cycling and apoptosis (ie. *OFS, MYC, MKP1, RANBP1, BAX, BIRC5, HMGA1, and CDK6*), immune response (*JUNB*, *NFKBIA*, *PSMB9*, *ZFP36*, *S100A10*, *IRF8*, *IL1B*, *FKBP1A*, and *VDAC1*), and differentiation (*S100A9* and *TNFSF13B*). **d**, Ligands expressed by $CD34^+$ HSPCs were ranked by likelihood that ligands would affect gene expression changes in $CD8^+$ T cells. Receptors expressed on $CD8^+$ T were selected based on their known potential to interact with prioritized ligands. Finally, target genes were selected based on their differential expression in $CD8^+$ T and their potential to be regulated by ligand-receptor interactions identified between $CD34^+$ HSPCs and $CD8^+$ T cells.

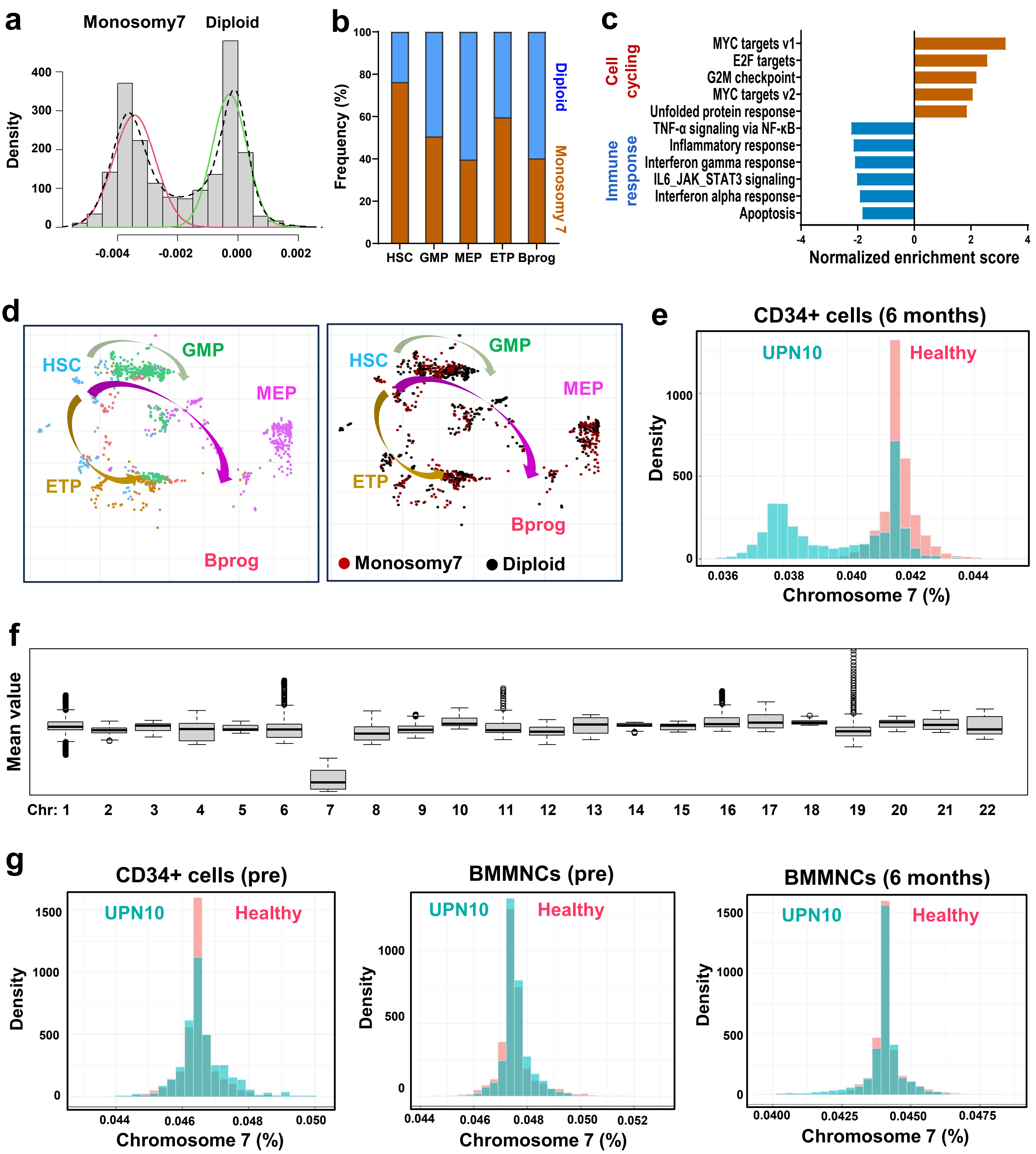

Supplementary Fig. 23 Detection of monosomy 7 cells by scRNA-seq. a, Histograms of read ratios on chromosome 7 in UPN10. Y-axis, density (frequency of cell number). X-axis, ratios of reads on chromosome 7 in individual cells relative to reads on all chromosomes of the same cell. **b**, Frequency (%) of monosomy 7 cells and diploid cells in each lineage in $CD34^+$ HSPCs of UPN10 at 6 months after treatment. **c**, Bar plot showing top pathways enriched in upregulated genes in monosomy 7 cells as compared to diploid cells in $CD34^+$ HSPCs of UPN10 at 6 months post-treatment. **d,** UMAPs of BMMNCs of UPN10 at 6 months after treatment, showing lineage differentiation (left), and distribution of monosomy 7 and diploid cells (right). **e**, Histograms of read ratios on chromosome 7 in $CD34^+$ cells of UPN10 at 6 months after treatment and in those of healthy donors. Green, cells from UPN10; pink, cells from healthy donors. y-axis, frequency of cell number; x-axis, ratios of reads on chromosome 7 in individual cells relative

to reads on all chromosomes in the same cells. **f**, Average gene expression for individual chromosomes in single $CD34^+$ cells of UPN10 at 6 months. Average gene expression levels of individual chromosomes from healthy donors were used for comparison. Chromosomal mapping read values were median centered. Top and bottom of the bars represent 25% and 75% quartiles, respectively. **g**, Histograms of read ratios on chromosome 7 in $CD34^+$ cells pretreatment, BMMNCs pretreatment, and BMMNCs at 6 months after treatment of UPN10. Green, cells from UPN10; pink, cells from healthy donors. y-axis, frequency of cell number; x-axis, ratios of reads on chromosome 7 in individual cells relative to reads on all chromosomes in the same cells.
