# Supplementary material for: Human autoimmunity at single cell resolution in aplastic anemia before and after effective immunotherapy
Source: Nat Commun. 2025 May 30;16:5048. doi: 10.1038/s41467-025-60213-6 (PMC12125301; doi:10.1038/s41467-025-60213-6)
Supplement: Supplementary file 2 — Description of Additional Supplementary Files [file 41467_2025_60213_MOESM2_ESM.pdf]

Description of Additional Supplementary Files

Supplementary Data 1. Patients' characteristics.

Supplementary Data 2. An antibody panel for CyTOF.

Supplementary Data 3. TCR sequences for GLIPH2 analysis.

Supplementary Data 4. Single-cell sequence metrics of single-cell RNA and single-cell TCR/BCR V(D)J Sequencing.
