# Supplementary material for: Human autoimmunity at single cell resolution in aplastic anemia before and after effective immunotherapy
Source: Nat Commun. 2025 May 30;16:5048. doi: 10.1038/s41467-025-60213-6 (PMC12125301; doi:10.1038/s41467-025-60213-6)
Supplement: Supplementary file 7 — Reporting Summary [file 41467_2025_60213_MOESM7_ESM.pdf]

nature portfolio

Corresponding author(s): Zhijie Wu

Last updated by author(s): Apr 7, 2025

Reporting Summary

Nature Portfolio wishes to improve the reproducibility of the work that we publish. This form provides structure for consistency and transparency in reporting. For further information on Nature Portfolio policies, see our Editorial Policies and the Editorial Policy Checklist.

Statistics

For all statistical analyses, confirm that the following items are present in the figure legend, table legend, main text, or Methods section.

n/a	Confirmed	
☐	☒	The exact sample size (n) for each experimental group/condition, given as a discrete number and unit of measurement
☐	☒	A statement on whether measurements were taken from distinct samples or whether the same sample was measured repeatedly
☐	☒	The statistical test(s) used AND whether they are one- or two-sided<br>*Only common tests should be described solely by name; describe more complex techniques in the Methods section.*
☒	☐	A description of all covariates tested
☐	☒	A description of any assumptions or corrections, such as tests of normality and adjustment for multiple comparisons
☐	☒	A full description of the statistical parameters including central tendency (e.g. means) or other basic estimates (e.g. regression coefficient) AND variation (e.g. standard deviation) or associated estimates of uncertainty (e.g. confidence intervals)
☐	☒	For null hypothesis testing, the test statistic (e.g. F, t, r) with confidence intervals, effect sizes, degrees of freedom and P value noted<br>*Give P values as exact values whenever suitable.*
☒	☐	For Bayesian analysis, information on the choice of priors and Markov chain Monte Carlo settings
☒	☐	For hierarchical and complex designs, identification of the appropriate level for tests and full reporting of outcomes
☐	☒	Estimates of effect sizes (e.g. Cohen's d, Pearson's r), indicating how they were calculated

Our web collection on statistics for biologists contains articles on many of the points above.

Software and code

Policy information about availability of computer code

Data collection	Single cell RNA sequencing (scRNA-seq) data was collected using an Illumina NovaSeq. Cytometry by Time-Of-Flight (CyTOF) data was collected using CyTOF-2 mass cytometer. Flow cytometry data was collected by BD Fortessa.
Data analysis	Flow cytometry data was analyzed by FlowJo (V10) software. Single cell RNA sequencing data was analyzed by 10X CellRanger7.0.1 package, available from 10x Genomics (https://support.10xgenomics.com/single-cell-gene-expression/software/pipelines/latest/what-is-cell-ranger), the Seurat R package (http://satijalab.org/seurat/, v4.3.0), fgsea R package and PhenoGraph Python package, AUCell package in Bioconductor, Monocle R package, tCR package, GenVisR (1.36.0 Bioconductor), miloR (1.8.1 Bioconductor), scvelo (0.2.4 https://github.com/theislab/scvelo), cbsniffer (https://github.com/sridnona/cb_sniffer), H-MAGMA (https://github.com/thewonlab/H-MAGMA), cellphoneDB (3.1.0 https://www.cellphonedb.org/), NicheNetR (2.0.2, https://github.com/saeyslab/nichenetr), infercnv (1.3.3, https://github.com/broadinstitute/infercnv), and WebLoGo (https://weblogo.berkeley.edu/logo.cgi). The code is available at Github: https://github.com/shouguog/SAA.

For manuscripts utilizing custom algorithms or software that are central to the research but not yet described in published literature, software must be made available to editors and reviewers. We strongly encourage code deposition in a community repository (e.g. GitHub). See the Nature Portfolio guidelines for submitting code & software for further information.

Data

Policy information about availability of data

All manuscripts must include a data availability statement. This statement should provide the following information, where applicable:

- Accession codes, unique identifiers, or web links for publicly available datasets
- A description of any restrictions on data availability
- For clinical datasets or third party data, please ensure that the statement adheres to our policy

The raw and analyzed sequencing data in this study have been deposited in the NCBI's Gene Expression Omnibus (under series accession code GSE247531) and Sequence Read Archive (under accession code PRJNA1039299). Source data are provided with this paper. A reporting summary for this article is available as a Supplementary Information file. Previously published data used for analysis in this study include: GSE101660 [https://www.ncbi.nlm.nih.gov/geo/query/acc.cgi?acc=GSE101660] and GSE168859 [https://www.ncbi.nlm.nih.gov/geo/query/acc.cgi?acc=GSE168859].

Research involving human participants, their data, or biological material

Policy information about studies with human participants or human data. See also policy information about sex, gender (identity/presentation), and sexual orientation and race, ethnicity and racism.

Reporting on sex and gender	Sex and gender was not considered in study design, nor was analyzed for results except showing in patients' characteristics. Sex was based on self-report but also on karyotyping testing.
Reporting on race, ethnicity, or other socially relevant groupings	Race, ethnicity, or other socially relevant grouping were not included, as they are not within the scope of the current study.
Population characteristics	There are 20 patients (9 female and 11 male, median age 36). They were all diagnosed as SAA. Details were listed in Extended Data Figure. 1. Clinical and laboratory characteristics of patients. There are 4 age- and sex-matched healthy donors (62/F, 57/M, 27/F, and 23/M). Bone marrow samples were obtained from these patients and healthy donors after written informed consent.
Recruitment	All participants were enrolled in research studies that had been approved approved by the Institutional Review Boards of the National Heart, Lung, and Blood Institute, in accordance with the Declaration of Helsinki, provided written informed consent. Clinical diagnoses were defined according to standard criteria. We conducted a prospective phase 2 study. A total of 139 subjects were enrolled: their SAA had not been definitively treated with ATG-based IST; and they lacked a suitable matched sibling marrow donor or were not candidates for hematopoietic stem cell transplantation, due to patients' choices, advanced ages, or socioeconomic factors. Patients, 2 years of age or older, were enrolled from December 2014 to May 2022 and treated with hATG, CSA and eltrombopag combination (hATG from day 1 for 4 days, therapeutic dosing of CSA from day 1 for 6 months, and eltrombopag starting from day 1 to 6 months). All patients met clinical criteria for SAA. Exclusion criteria were as previously reported. For subjects who met the criteria for very SAA (VSAA; absolute neutrophil count (ANC) <= 200 x 109/l), treatment was initiated based on morphologic confirmation while cytogenetic studies were pending.
Ethics oversight	Written informed consent were under protocol (www.clinicaltrials.gov NCT01623167) approved by the Institutional Review Boards of National Heart, Lung, and Blood Institute, in accordance with the Declaration of Helsinki.

Note that full information on the approval of the study protocol must also be provided in the manuscript.

Field-specific reporting

Please select the one below that is the best fit for your research. If you are not sure, read the appropriate sections before making your selection.

☒ Life sciences ☐ Behavioural & social sciences ☐ Ecological, evolutionary & environmental sciences

For a reference copy of the document with all sections, see nature.com/documents/nr-reporting-summary-flat.pdf

Life sciences study design

All studies must disclose on these points even when the disclosure is negative.

Sample size	Sample size for the current exploratory laboratory study was arbitrarily determined to be 20. No sample size calculation was performed. Bone marrow samples before and after treatment were obtained from 20 SAA patients after written informed consent under clinical protocol. In total, ~787,568 BMMNCs and 566,528 FACS sorted CD34+ HSPCs were sequenced from these 20 patients and 4 healthy controls.
Data exclusions	Sequencing data were processed and filtered using well-established pipeline, and only single cells passing quality control were retained for further analysis.
Replication	Biological replication dose not apply to human samples, instead, we included multiple individuals in each group for comparison (20 individuals in patient group and 4 individuals in healthy control group). No technical replication was performed due to cost of experiments.
Randomization	The original clinical protocol (NCT01623167) was designed as a prospective phase 2 study that enrolls patients with severe aplastic anemia to be treated with hATG, CSA and EPAG.

Blinding	The original clinical protocol (NCT01623167) was designed as a prospective phase 2 study that enrolls patients with severe aplastic anemia (SAA) to be treated with horse antithymocyte globulin (h-ATG), cyclosporine (CSA) and eltrombopag (EPAG). There is a single arm and blinding is not applicable.

Reporting for specific materials, systems and methods

We require information from authors about some types of materials, experimental systems and methods used in many studies. Here, indicate whether each material, system or method listed is relevant to your study. If you are not sure if a list item applies to your research, read the appropriate section before selecting a response.

Materials & experimental systems

n/a	Involved in the study	
☐	☒	Antibodies
☒	☐	Eukaryotic cell lines
☒	☐	Palaeontology and archaeology
☒	☐	Animals and other organisms
☐	☒	Clinical data
☐	☒	Dual use research of concern
☒	☐	Plants

Methods

n/a	Involved in the study	
☒	☐	ChIP-seq
☐	☒	Flow cytometry
☒	☐	MRI-based neuroimaging

Antibodies

Antibodies used

We described antibodies used in this study in the Supplementary Materials and Methods
1. Pacific Blue™ anti-human Lineage Cocktail (CD3, CD14, CD16, CD19, CD20, CD56), Biolegend, Catalog#348805.
2. CD34 Mouse anti-Human, PE, Clone: 581, BD, Catalog#555822;
3. APC Mouse Anti-Human CD38, Clone HIT2 (RUO), Catalog#555462;
4. FITC anti-human CD90 (Thy1) Antibody, Clone 5E10, Catalog#328108;
5. BV605 Mouse Anti-Human CD10, Clone HI10a (RUO), Catalog#562978;
6. PE/Cyanine7 anti-human CD135 (Flt-3/Flk-2) Antibody, Clone BV10A4H2, Catalog#313314;
7. Brilliant Violet 510™ anti-human CD45RA Antibody, Clone HI100, Catalog#304142.

Antibodies below were used for CyTOF.

8. Anti-Human CD45 (HI30)-89Y, Catalog#3089003B.
9. Purified anti-human CD68 Antibody, Clone Y1/82A, Catalog#333802.
10. Fluidigm Corp Anti-Human CD19-142RN, Clone HIB19, Catalog#3142001B.
11. Anti-Human CD45 (HI30)-198Pt, Clone HI100, Catalog#3143006B.
12. Anti-Human CD15/SSEA-1 (W6D3)-144Nd, Clone W6D3, Catalog#3144019B.
13. Anti-Human CD4 (RPA-T4)-145Nd, Clone RPAT4, Catalog#3145001B.
14. Anti-Human CD8a (C8/144B)-162Dy, Clone RPAT8, Catalog#3146001B.
15. Anti-Human CD11c (Bu15)-147Sm, Clone Bu15, Catalog#3147008B.
16. Purified anti-human CD235ab (Maxpar® Ready) Antibody, Clone HIR2, Catalog#306615.
17. Anti-Human CD194/CCR4 (L291H4)-149Sm, Clone L291H4, Catalog#3149029A.
18. Anti-Human CD61 (VI-PL2)-146Nd, Clone VI-PL2, Catalog#3150001B.
19. Anti-Human CD103 (Ber-ACT8)-151Eu, Clone BerACT8, Catalog#3151011B.
20. Anti-Human CD95/Fas (DX2)-152Sm, Clone DX2, Catalog#3152017B.
21. Purified anti-human CD161 antibody, Clone HP-3G10, Catalog#339919.
22. Anti-Human CD3 (Polyclonal, C-Terminal)-170Er, Clone UCTH1, Catalog#3154003B.
23. ANTI HUMAN CD27 L128 155GD, Clone L128, Catalog#3155001B.
24. Anti-Human CD169/Siglec-1 (7-239)-158Gd, Clone 7-239, Catalog#3158027B.
25. Anti-Human CD197/CCR7 (G043H7)-159Tb, Clone G043H7, Catalog#3159003A.
26. ANTI HUMAN CD39 A1 160GD, Clone A1, Catalog#3160004B.
27. Anti-Human CD49b (P1E6-C5)-161Dy, Clone P1E6-C5, Catalog#3161012B.
28. Anti-Human CD66b (80H3)-162Dy, Clone 80H3, Catalog#3162023B.
29. CD33 WM53 Fluidigm 3163023B, Clone WM53, Catalog#3163023B.
30. Anti-Human CD45RO (UCHL1)-149Sm, Clone UCHL1, Catalog#3164007B.
31. Anti-Human CD223/LAG-3 (11C3C65)-165Ho, Clone 11C3C65, Catalog#3165037B.
32. Anti-Human CD34 (581)-166Er, Clone 581, Catalog#3166012B.
33. Anti-Human CD11b/Mac-1 (ICRF44)-144Nd, Clone ICRF44, Catalog#3167011B.
34. Anti-Human CD127/IL-7Ra (A019D5)-168Er, Clone A019D5, Catalog#3168017B.
35. Anti-Human CD25 (2A3)-149Sm, Clone 2A3, Catalog#3169003B.
36. Anti-Human CD25 Antibody M-A251, Clone MA-251, Catalog#356102.
37. Anti-Human HLA-DR (L243)-143Nd, Clone L243, Catalog#3170013B.
38. Purified anti-mouse/rat/human FOXP3 Antibody, Clone 150D, Catalog#320002.
39. Purified anti-human FOXP3 Antibody, Clone 259D, Catalog#320202.
40. FOXP3 Monoclonal Antibody (PCH101), Clone PCH101, Catalog#14-4776-82.

41. Anti-Human CD38 (EPR4106)-141Pr, Clone HIT2, Catalog#3172007B.
42. Anti-Human CD184/CXCR4 (12G5)-156Gd, Clone 12G5, Catalog#3173001B.
43. Anti-Human CD279/PD-1 (EH12.2H7)-165Ho, Clone EH12.2H7, Catalog#3174020B.
44. Anti-Human CD14 M5E2, Clone M5E2, Catalog#3175015B.
45. Anti-Human CD57 (HCD57)-176Yb, Clone HCD57, Catalog#3176019B.
46. Anti-Human CD16 (3G8)-209Bi, Clone 3G8, Catalog#3209002B.

Validation

1. https://www.biolegend.com/it-it/products/pacific-blue-anti-human-lineage-cocktail-cd3-cd14-cd16-cd19-cd20-cd56-8250
2. https://www.fishersci.com/shop/products/cd34-mouse-anti-human-pe-clone-581-bd/BDB555822
3. https://www.bdbiosciences.com/en-eu/products/reagents/flow-cytometry-reagents/research-reagents/single-color-antibodies-ruo/apc-mouse-anti-human-cd38.555462
4. https://www.biolegend.com/it-it/products/fitc-anti-human-cd90-thy1-antibody-4113
5. https://www.bdbiosciences.com/en-ca/products/reagents/flow-cytometry-reagents/research-reagents/single-color-antibodies-ruo/bv605-mouse-anti-human-cd10.562978
6. https://www.biolegend.com/it-it/products/pe-cyanine7-anti-human-cd135-flt-3-flk-2-antibody-11672
7. https://www.biolegend.com/it-it/products/brilliant-violet-510-anti-human-cd45ra-antibody-8007

Antibodies below were used for CyTOF.

8. https://store.standardbio.com/product_detail/guest-catalog/3089003b/
9. https://www.biolegend.com/de-de/products/purified-anti-human-cd68-antibody-4835
10. https://www.fishersci.com/shop/products/anti-hu-cd19-142nd-1/50466788
11. https://www.standardbio.com/resources/safety-data-sheets?&page=184
12. https://www.citeab.com/antibodies/12121855-3144019b-anti-human-cd15-ssea-1-w6d3-144nd-100-te
13. https://www.citeab.com/antibodies/12122071-3145001b-anti-human-cd4-rpa-t4-145nd-100-tests
14. https://www.standardbio.com/resources/safety-data-sheets?&page=219
15. https://www.citeab.com/antibodies/12122045-3147008b-anti-human-cd11c-bu15-147sm-100-tests
16. https://www.biolegend.com/en-gb/products/purified-anti-human-cd235ab-maxpar-ready-antibody-9192
17. https://www.standardbio.com/resources/safety-data-sheets?&page=116
18. https://www.standardbio.com/resources/safety-data-sheets?&page=201
19. https://www.standardbio.com/resources/safety-data-sheets?&page=70
20. https://www.standardbio.com/sds/filter?&page=112
21. https://www.biolegend.com/fr-ch/products/purified-anti-human-cd161-maxpar-ready-antibody-9203
22. https://www.standardbio.com/resources/safety-data-sheets?&page=151
23. https://www.govsci.com/product-detail/Fluidigm/3155001B/EA/\
24. https://www.standardbio.com/sds/filter?&page=53
25. https://www.citeab.com/antibodies/12121596-3159003a-anti-human-cd197-ccr7-g043h7-159tb-50-te
26. https://www.govsci.com/product-detail/Fluidigm/3160004B/EA/
27. https://www.fishersci.com/shop/products/anti-human-cd49b-p1e6-c5-161/NC1627966
28. https://store.standardbio.com/product_detail/guest-catalog/3162023b/
29. https://www.fishersci.com/shop/products/anti-hum-cd33-wm53-163dy-100t/NC1460532
30. https://www.standardbio.com/sds/filter?&page=94
31. https://www.fishersci.com/shop/products/anti-human-cd223-lag-3-11c3c6/NC2298074
32. https://www.citeab.com/antibodies/12122243-3166012b-anti-human-cd34-581-166er-100-tests
33. https://www.standardbio.com/resources/safety-data-sheets?&page=74
34. https://www.citeab.com/antibodies/12122400-3168017b-anti-human-cd127-il-7ra-a019d5-168er-100
35. https://www.standardbio.com/resources/safety-data-sheets?&page=135
36. https://www.biolegend.com/fr-ch/products/apc-anti-human-cd25-antibody-8452
37. https://www.standardbio.com/sds/filter?&page=119
38. https://www.biolegend.com/fr-ch/products/purified-anti-mouse-rat-human-foxp3-antibody-2886
39. https://www.biolegend.com/fr-lu/products/purified-anti-human-foxp3-antibody-2903
40. https://www.thermofisher.com/antibody/product/FOXP3-Antibody-clone-PCH101-Monoclonal/14-4776-82
41. https://www.standardbio.com/sds/filter?&page=85
42. https://www.standardbio.com/resources/safety-data-sheets?&page=109
43. https://www.standardbio.com/sds/filter?&page=74
44. https://www.govsci.com/product-detail/Fluidigm/3175015B/EA/
45. https://www.citeab.com/antibodies/12121756-3176019b-anti-human-cd57-hcd57-176yb-100-tests
46. https://store.standardbio.com/product_detail/guest-catalog/3209002b/

Clinical data

Policy information about clinical studies

All manuscripts should comply with the ICMJEguidelines for publication of clinical research and a completedCONSORT checklist must be included with all submissions.

Clinical trial registration	www.clinicaltrials.gov NCT01623167
Study protocol	www.clinicaltrials.gov NCT01623167
Data collection	Patients were enrolled from December 2014 to May 2022 and treated with hATG, CSA and eltrombopag combination (hATG from day 1 for 4 days, therapeutic dosing of CSA from day 1 for 6 months, and eltrombopag starting from day 1 to 6 months), at hematology branch, NHLBI/NIH at Bethesda, MD, USA. Patients were followed up and re-evaluated, and also data collection at these time points at the hematology branch, NHLBI/NIH.
Outcomes	These patients diagnosed with SAA were treated with h-ATG, CSA, and EPAG combination. Patients were followed up for a median of 24.4 months. Primary efficacy endpoint is hematologic response at 6 months. Responders were included in secondary endpoint analysis of long-term outcomes. Secondary endpoints of this study included: (1) rates of relapse in patients deemed responders at 6 months, (2) clonal evolution to a myeloid malignancy or new chromosomal abnormality, (3) overall survival (OS), (4) hematologic response of relapsed subjects that restarted treatment, and (5) effects of CSA maintenance from 6 to 24 months on the rates of relapse.

Plants

Seed stocks	Not applicable.
Novel plant genotypes	Not applicable.
Authentication	Not applicable.

Flow Cytometry

Plots

Confirm that:

- [x] The axis labels state the marker and fluorochrome used (e.g. CD4-FITC).
- [x] The axis scales are clearly visible. Include numbers along axes only for bottom left plot of group (a 'group' is an analysis of identical markers).
- [x] All plots are contour plots with outliers or pseudocolor plots.
- [x] A numerical value for number of cells or percentage (with statistics) is provided.

Methodology

Sample preparation	BM specimens were obtained from patients and healthy donors, and processed within 6 hours after collection. BMMNCs were isolated from each person by density centrifugation using LSM Lymphocyte Separation Medium (Cat# 50494X, MP Biomedicals). Briefly, BM was diluted twofold using phosphate buffered saline (PBS) (Cat# 17-516Q, Lonza), layered on top of 1 volume LSM Lymphocyte Separation Medium in a 50-ml Falcon tube, and spun down at 1140g for 25 min at room temperature with brake off. A BMMNC layer was isolated and washed with PBS after red blood cell lysing with ACK lysing buffer (Cat# 118-156-101, Quality Biological). BMMNCs were resuspended in the IMDM (Cat# 12440053, Thermo Fisher Scientific) + 2% fetal bovine serum (Cat# 12306C, Sigma-Aldrich) before fluorescence-activated cell sorting (FACS) to enrich lineage-CD34+ hematopoietic stem and progenitor cells (HSPCs). BMMNCs were stained with monoclonal antibodies for 30 min on ice: anti-human lineage cocktail (CD3, CD14, CD16, CD19, CD20 and CD56; clones UCHT1, HCD14, 3G8, HIB19, 2H7 and HCD56, respectively, Cat# 348805, Biolegend) in Pacific Blue; anti-CD34 Ab (clone 581, Cat# 555822, BD Biosciences) in PE and anti-CD38Ab (clone HIT2, Cat# 555462, BD Biosciences) in APC. Cells were sorted using the FACSAria Fusion Flow Cytometer (BD Biosciences). Aliquots of BMMNCs were subjected to multi-color flow cytometry to profile HSP subpopulations. BMMNCs and purified lineage-CD34+ cells were subjected to scRNA-seq.
Instrument	BD LSR Fortessa cytometer
Software	Data were analyzed using FlowJo software (Tree Star Inc.)
Cell population abundance	Purity of sorted lineage-CD34+ cells were determined by flow cytometry with >95% purity.

Gating strategy

Cells were first plotted on FSC (x-axis) and SSC (y-axis) plot, gated for single cells, then lineage cocktail expression (CD3, CD14, CD16, CD19, CD20, and CD56) and CD34 expression level was used to determined Lineage-CD34+ HSPCs. Lineage-CD34+ population was further gated to Lineage-CD34+CD38- (HSC) and Lineage-CD34+CD38+ populations. Within Lineage-CD34+CD38+ population, cells were gated based on CD10 and CD45RA expression for CMP/MEP (Lineage-CD34+CD38+CD10-CD45RA-), GMP (Lineage-CD34+CD38+CD10-CD45RA+), and lymphoid progenitors LymP (Lineage-CD34+CD38+CD10+).

☒ Tick this box to confirm that a figure exemplifying the gating strategy is provided in the Supplementary Information.
